# Supplementary figures and images for: CRISPR-RfxCas13d screening uncovers Bckdk as a post-translational regulator of maternal-to-zygotic transition in teleosts
Source: EMBO J. 2025 Nov 18;44(23):7021–59. doi: 10.1038/s44318-025-00617-8 (PMC12669676; doi:10.1038/s44318-025-00617-8)

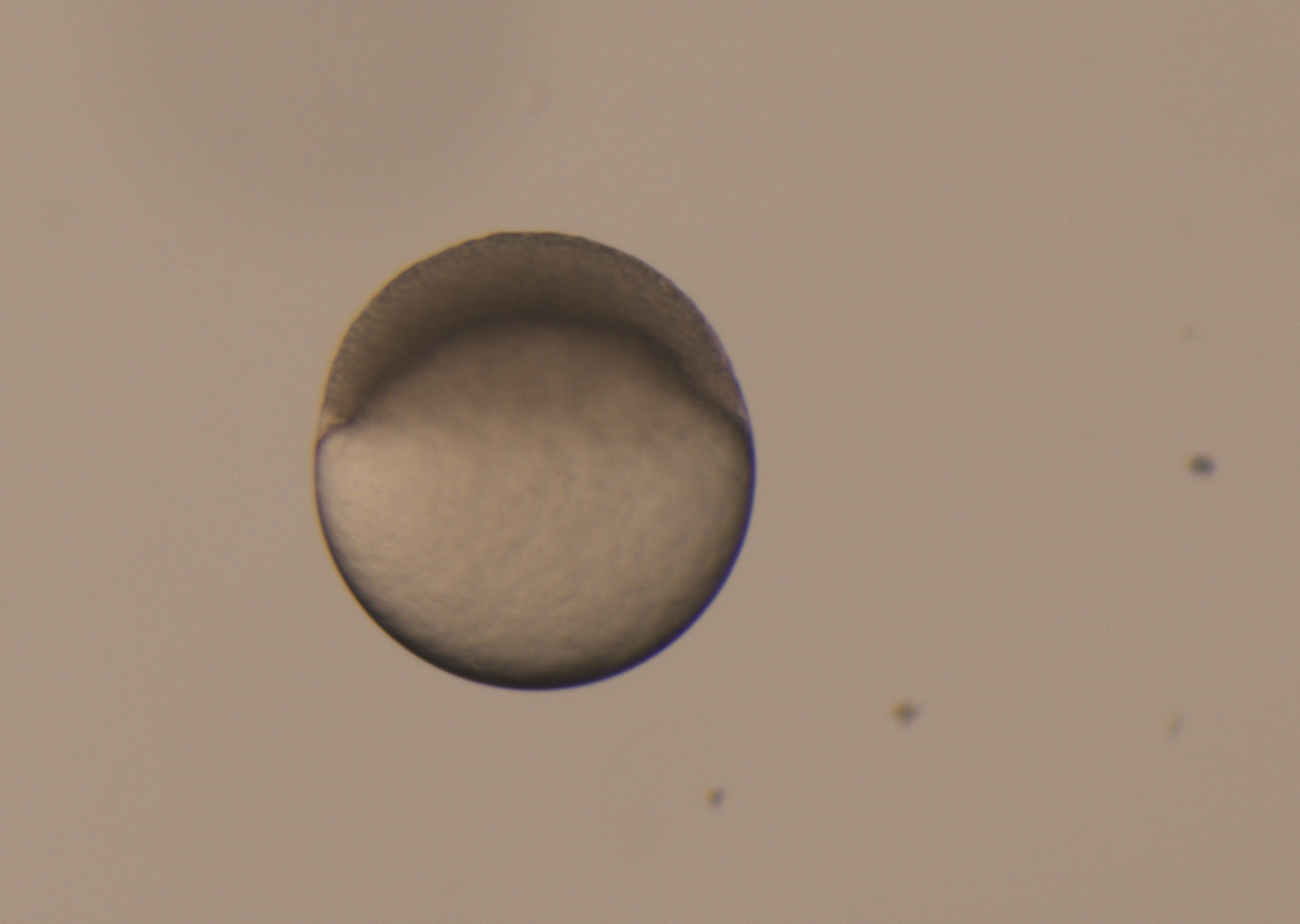

Supplement: Supplementary file 4 — Source data Fig. 1 [file 44318_2025_617_MOESM4_ESM.zip › Images_1C/30%epiboly.tif]

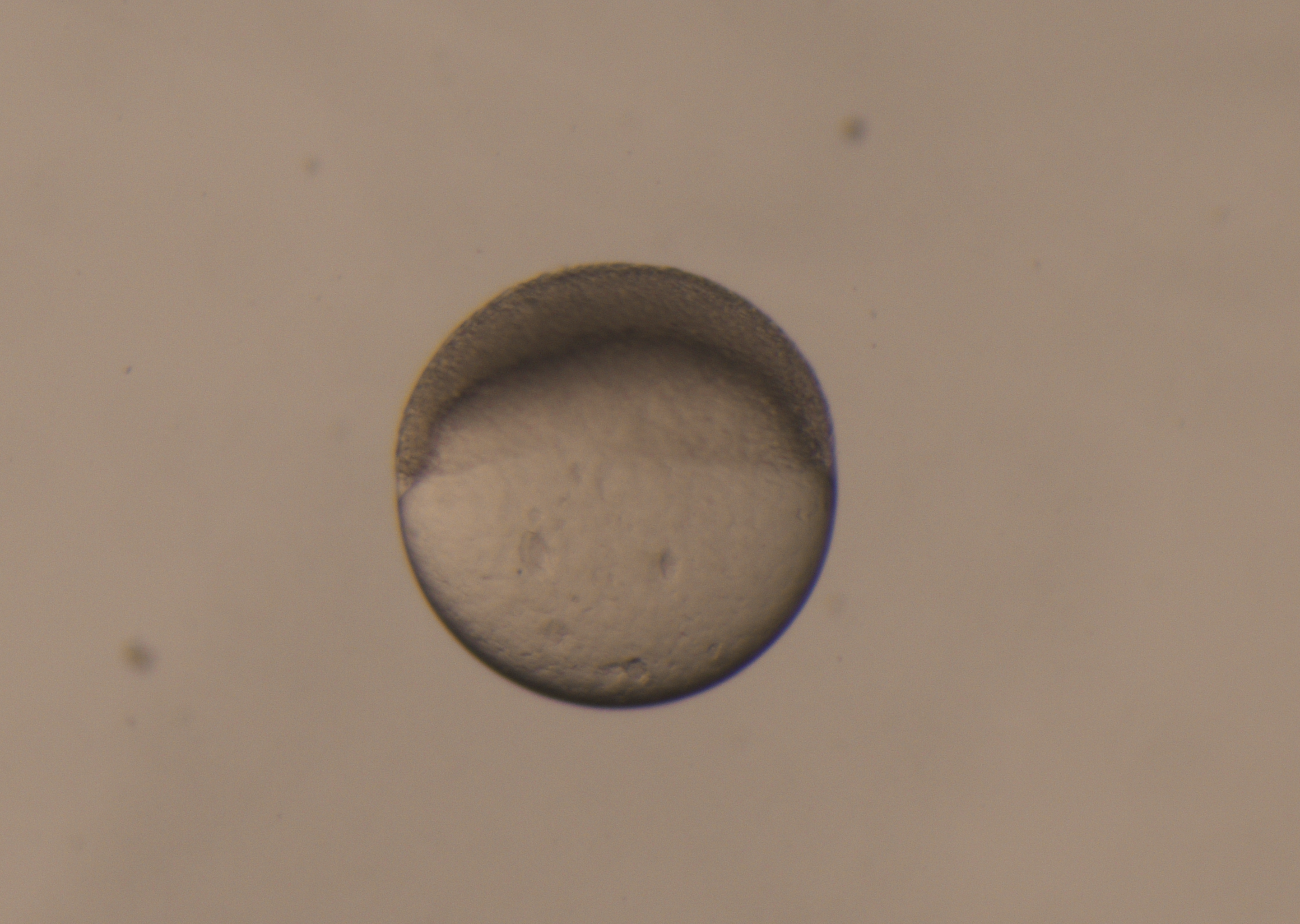

Supplement: Supplementary file 4 — Source data Fig. 1 [file 44318_2025_617_MOESM4_ESM.zip › Images_1C/50%epiboly.tif]

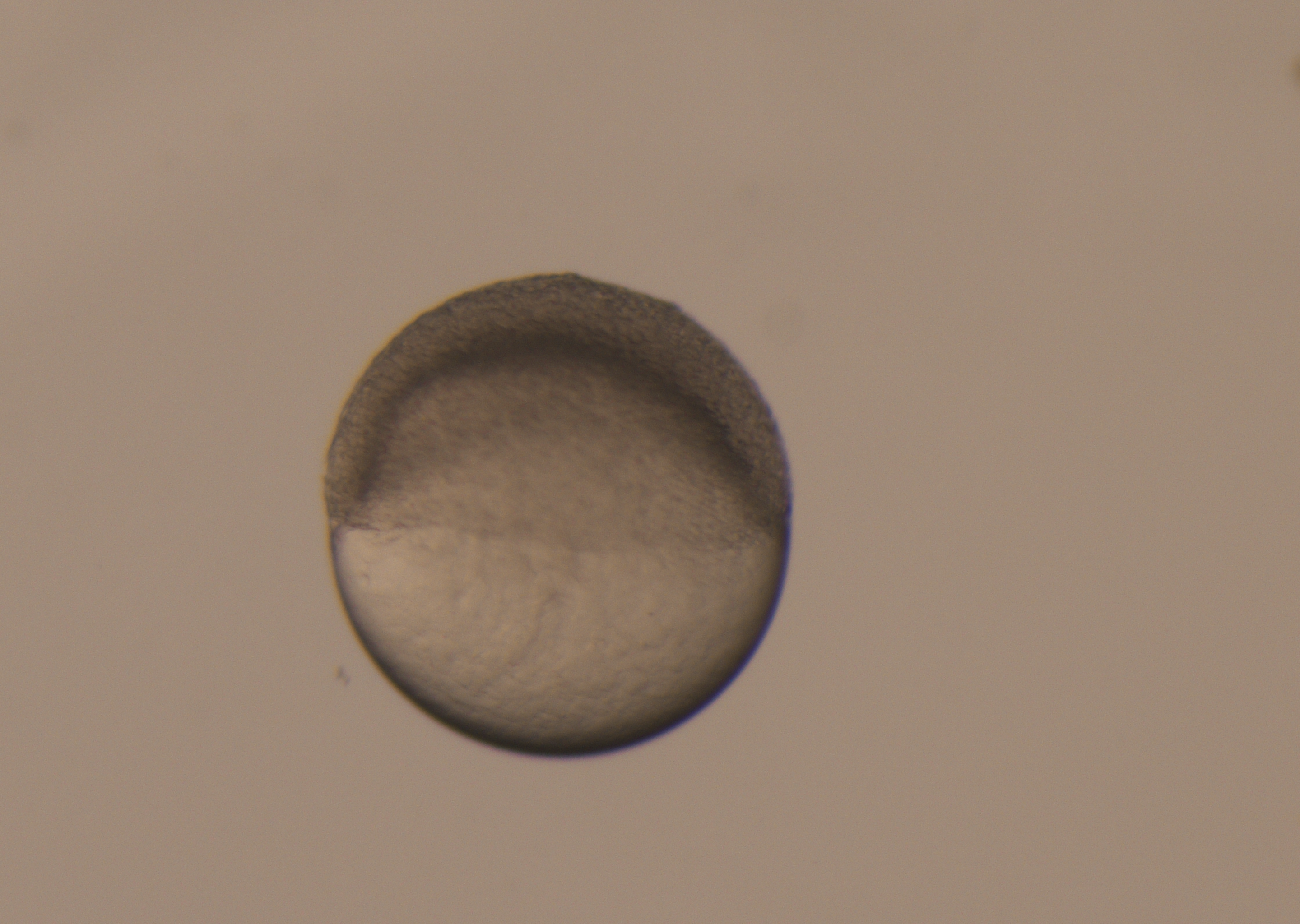

Supplement: Supplementary file 4 — Source data Fig. 1 [file 44318_2025_617_MOESM4_ESM.zip › Images_1C/germ_ring.tif]

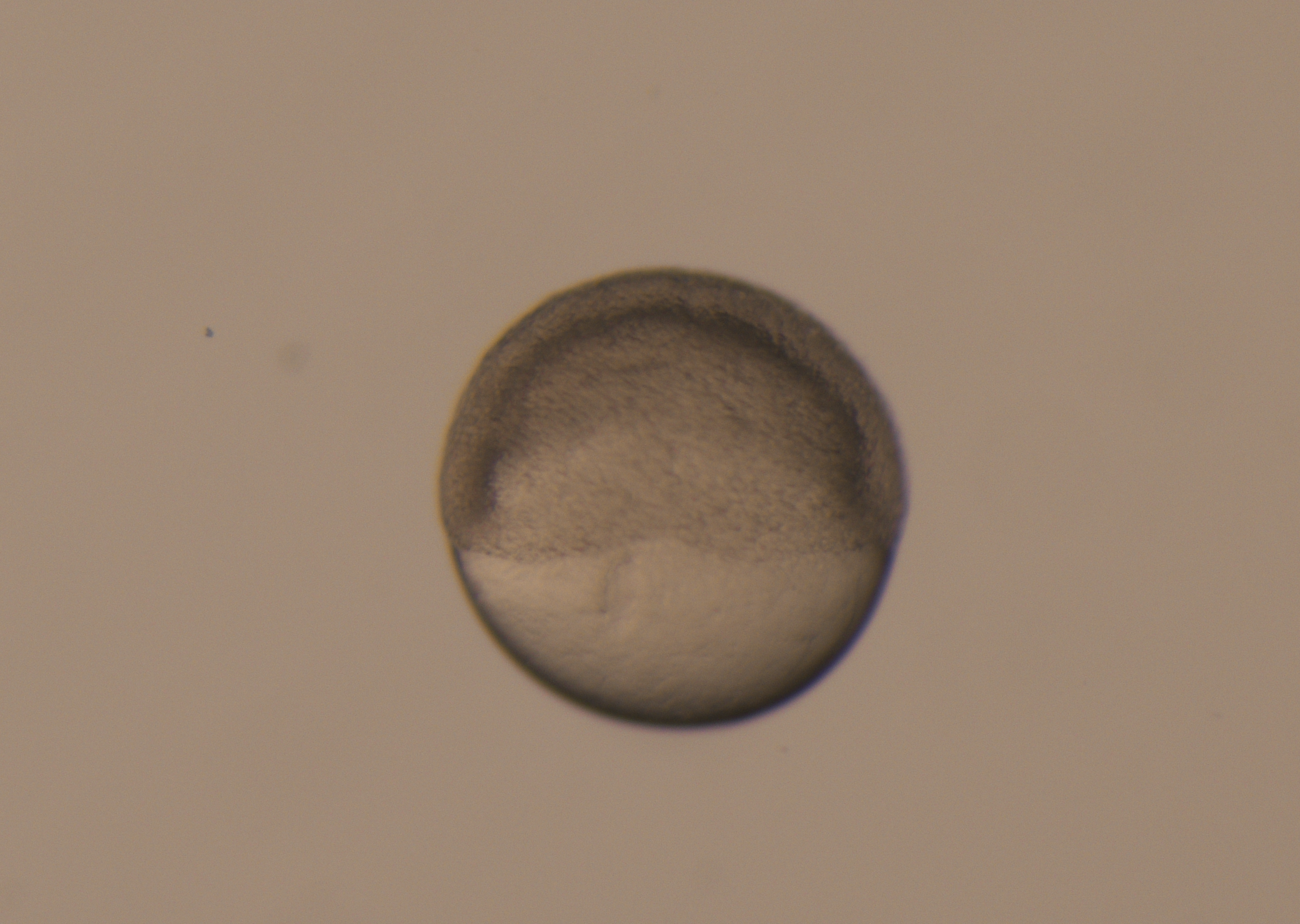

Supplement: Supplementary file 4 — Source data Fig. 1 [file 44318_2025_617_MOESM4_ESM.zip › Images_1C/shield.tif]

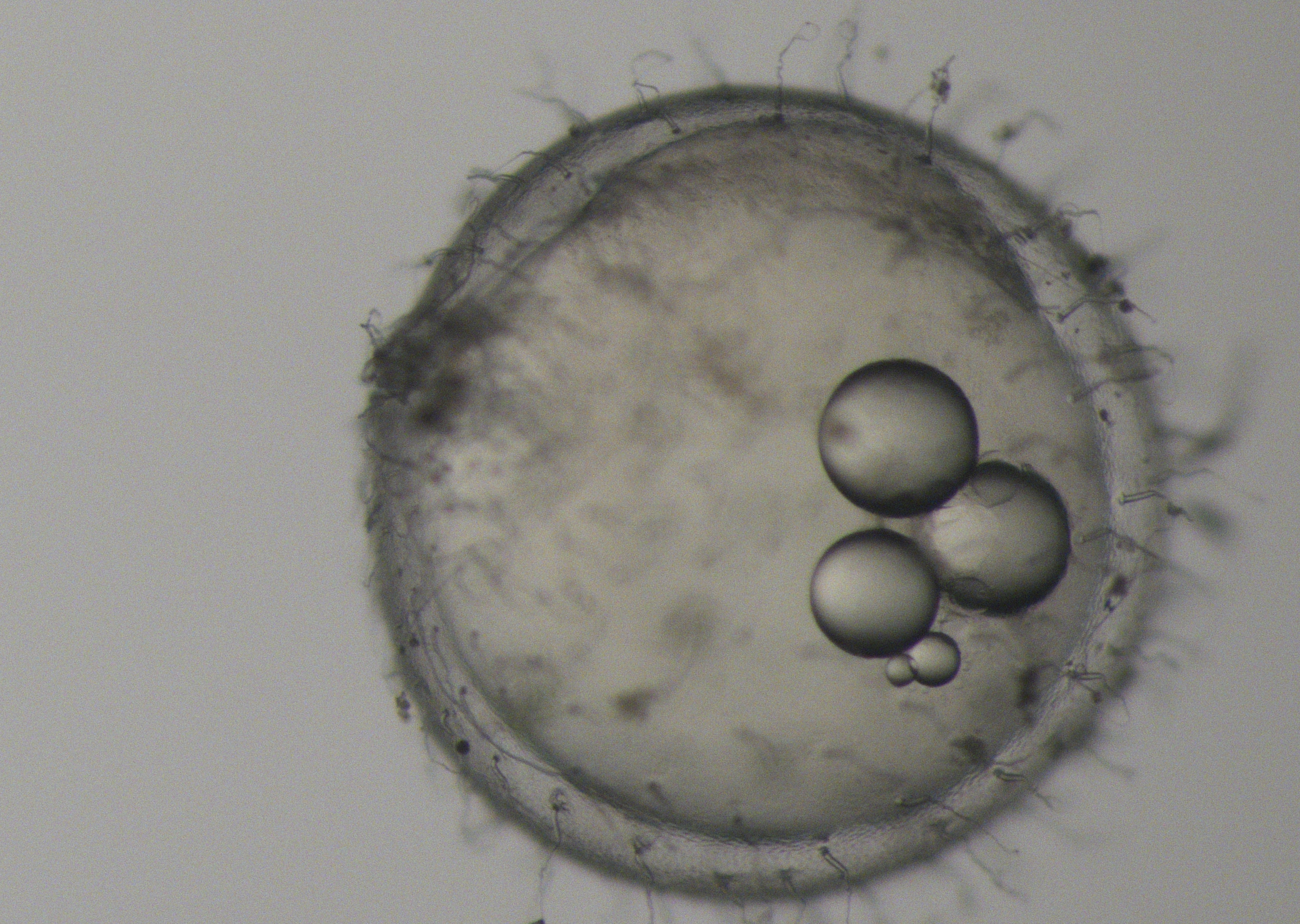

Supplement: Supplementary file 5 — Source data Fig. 2 [file 44318_2025_617_MOESM5_ESM.zip › Images_2G/stage_12.tif]

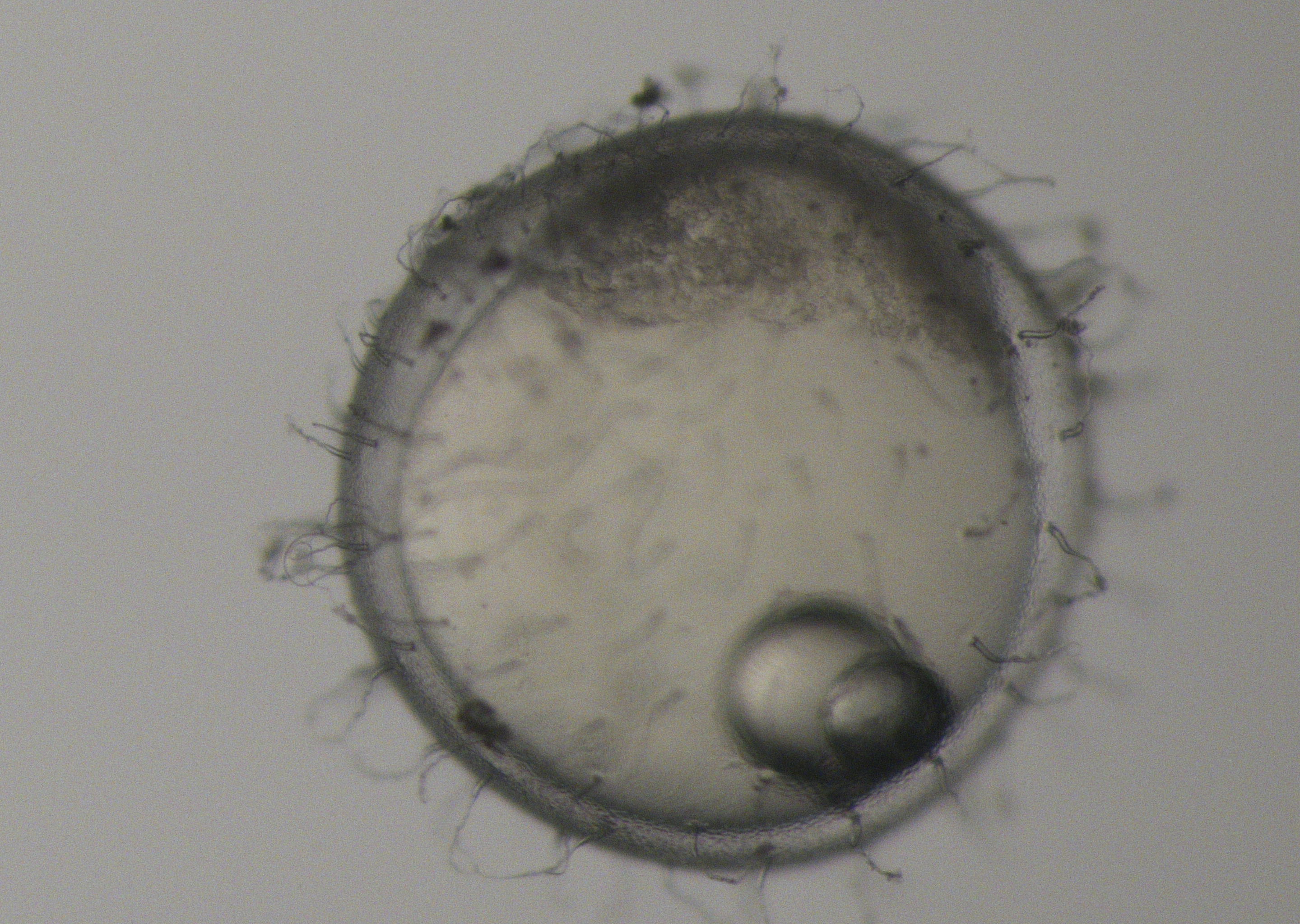

Supplement: Supplementary file 5 — Source data Fig. 2 [file 44318_2025_617_MOESM5_ESM.zip › Images_2G/stage_13.tif]

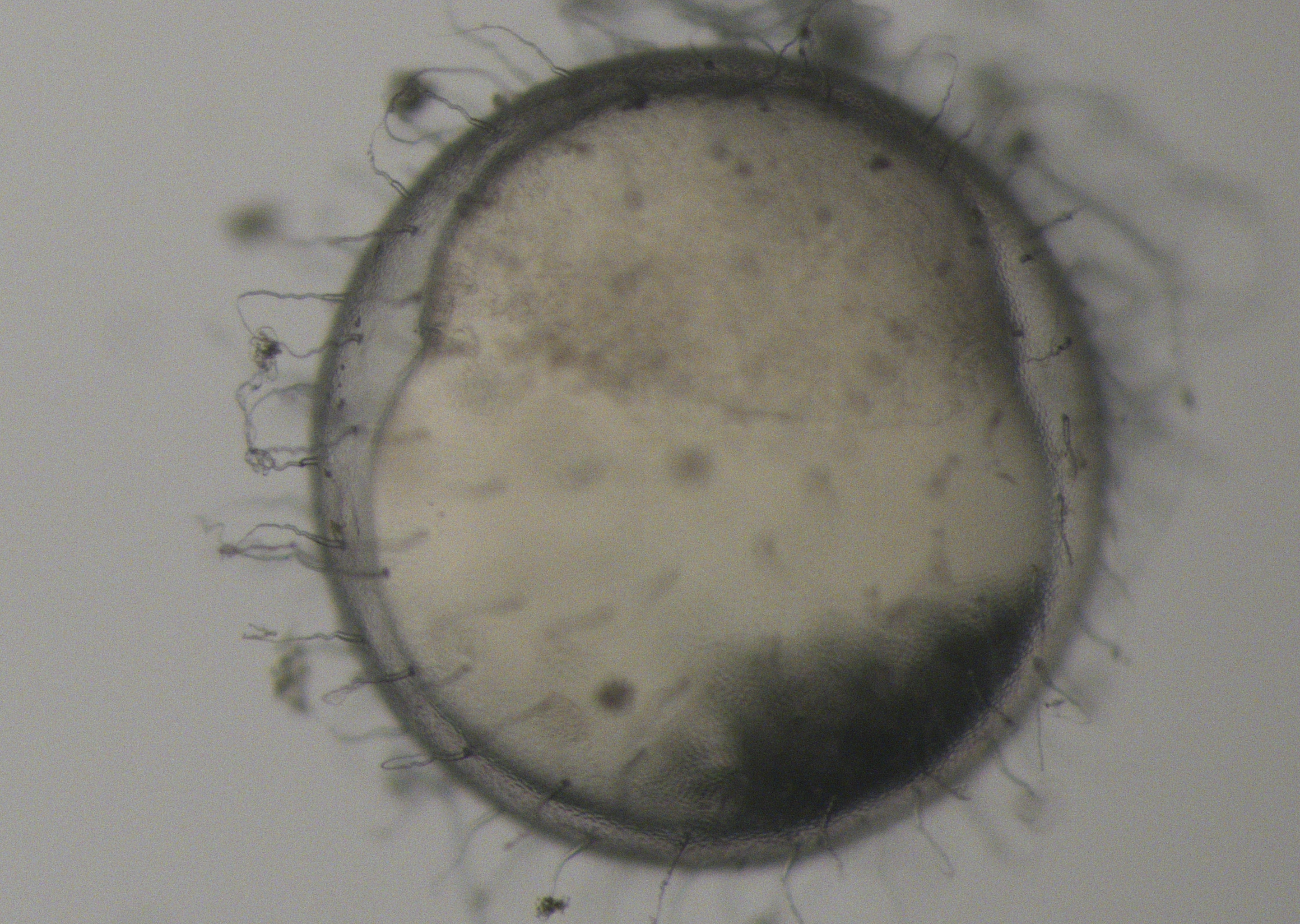

Supplement: Supplementary file 5 — Source data Fig. 2 [file 44318_2025_617_MOESM5_ESM.zip › Images_2G/stage_14.tif]

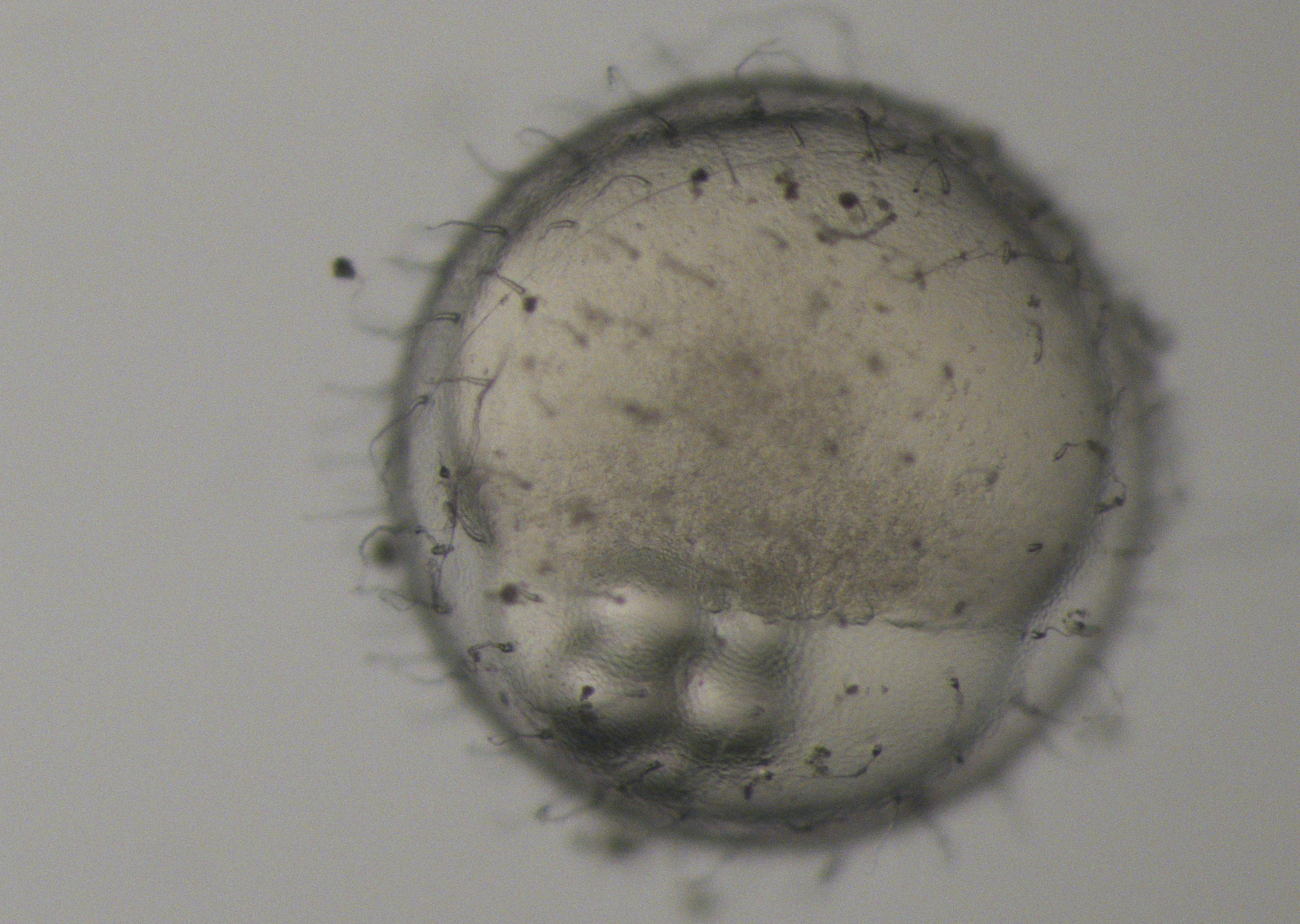

Supplement: Supplementary file 5 — Source data Fig. 2 [file 44318_2025_617_MOESM5_ESM.zip › Images_2G/stage_15.tif]

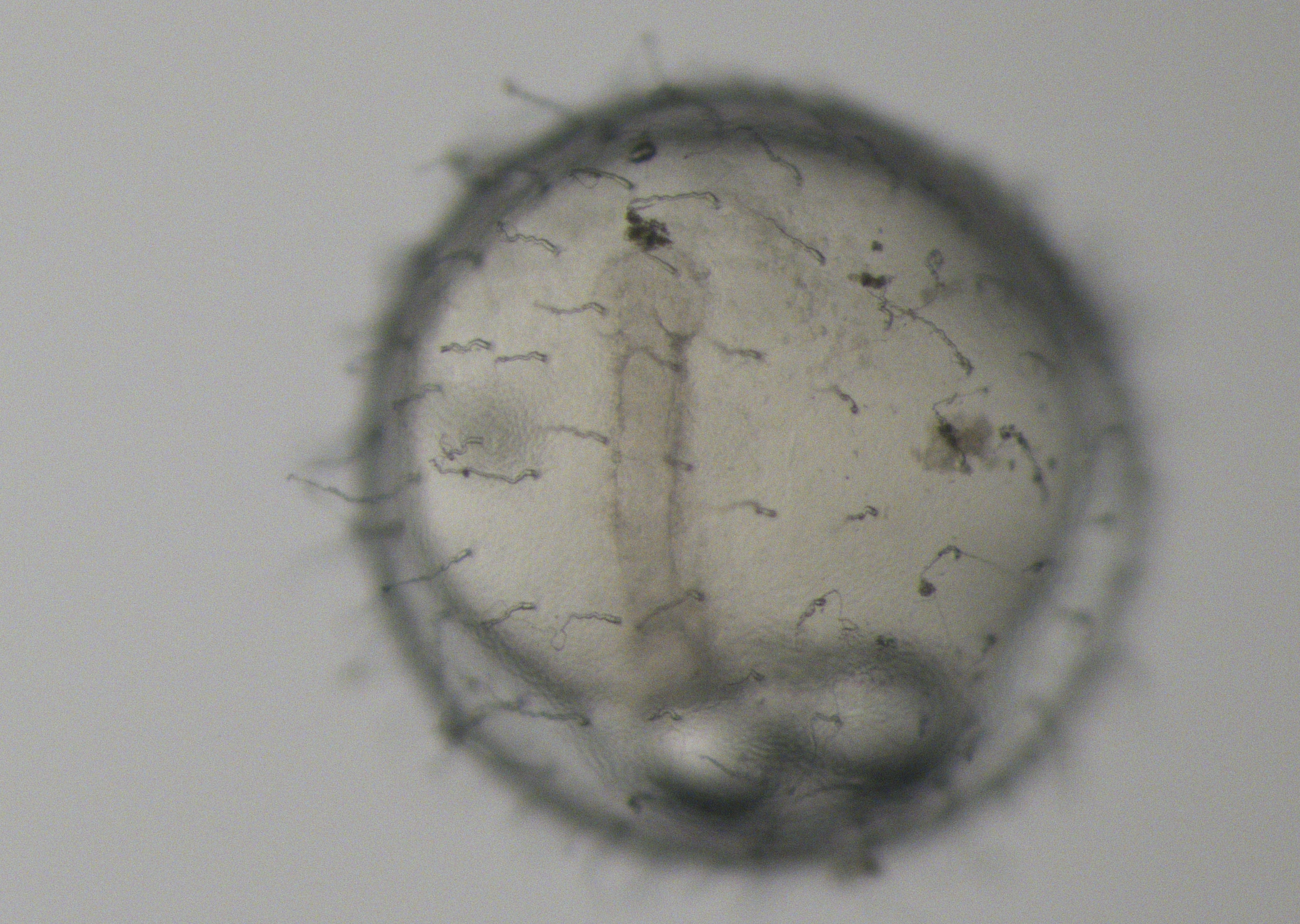

Supplement: Supplementary file 5 — Source data Fig. 2 [file 44318_2025_617_MOESM5_ESM.zip › Images_2G/stage_16.tif]

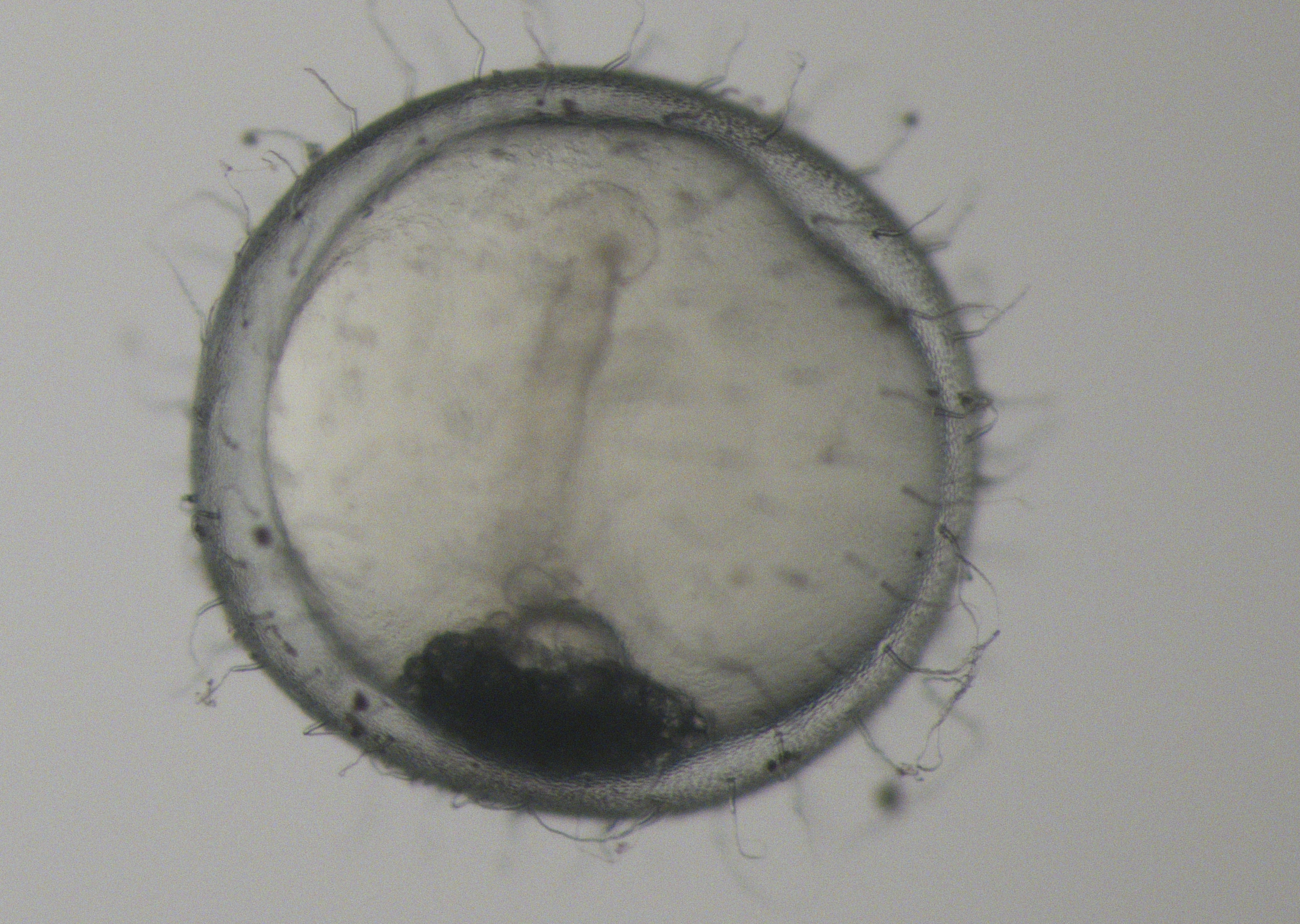

Supplement: Supplementary file 5 — Source data Fig. 2 [file 44318_2025_617_MOESM5_ESM.zip › Images_2G/stage_17.tif]

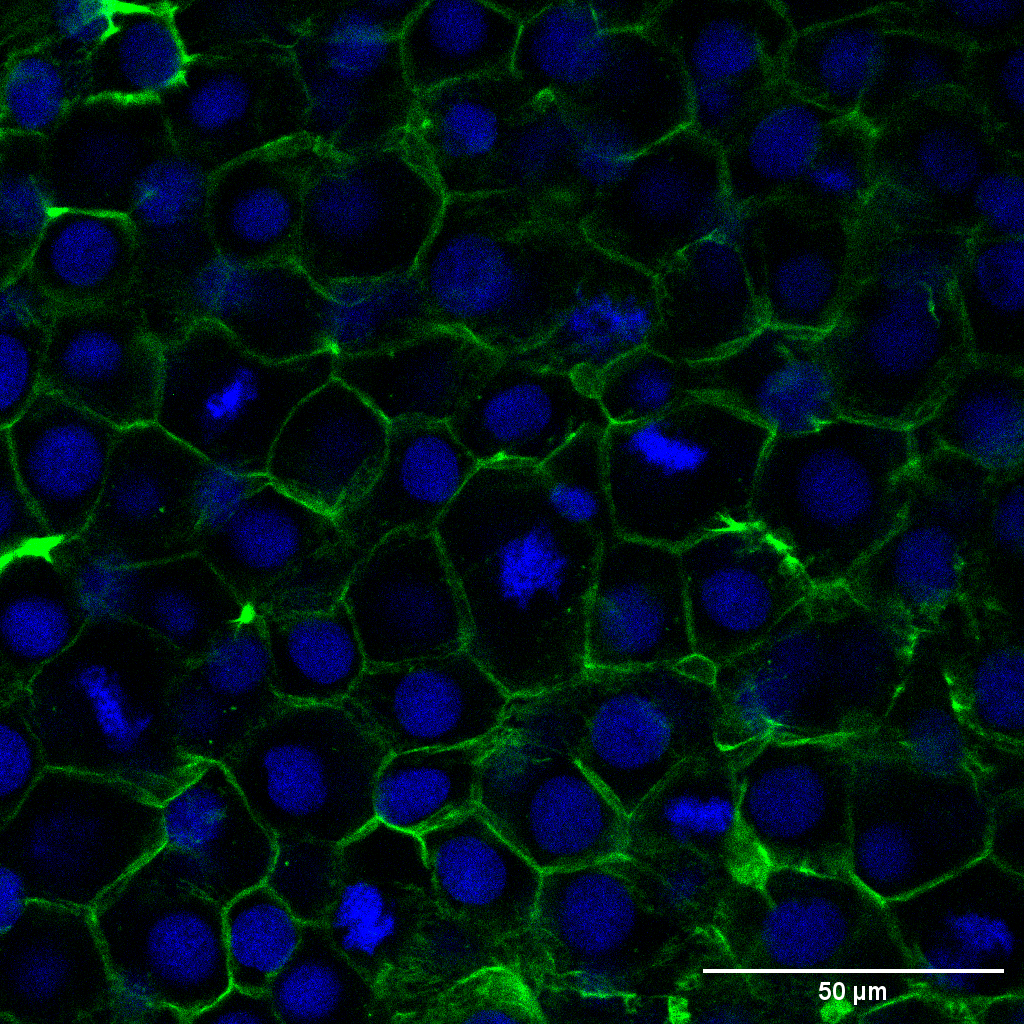

Supplement: Supplementary file 9 — EV Figure Source Data [file 44318_2025_617_MOESM9_ESM.zip › Images_EV5J/Bckdk_KD.png]

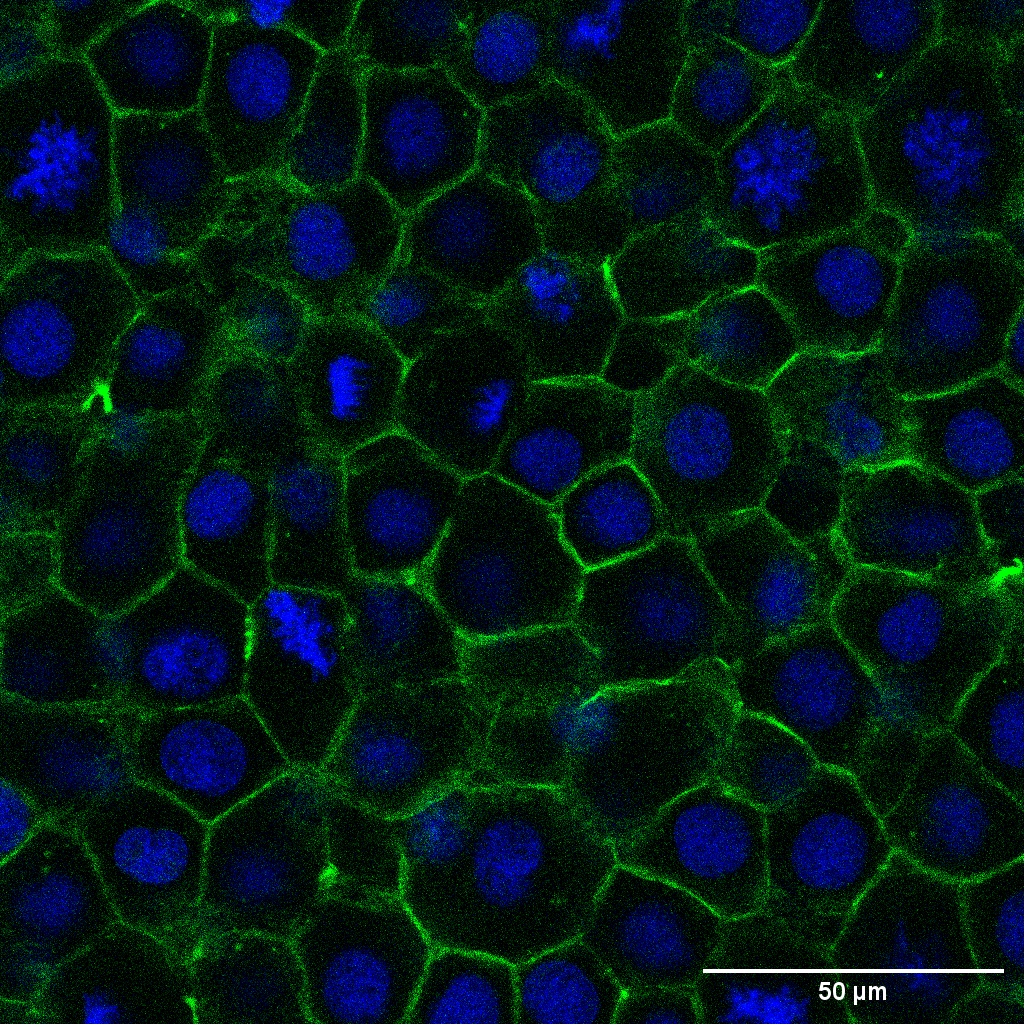

Supplement: Supplementary file 9 — EV Figure Source Data [file 44318_2025_617_MOESM9_ESM.zip › Images_EV5J/Cas13d.png]

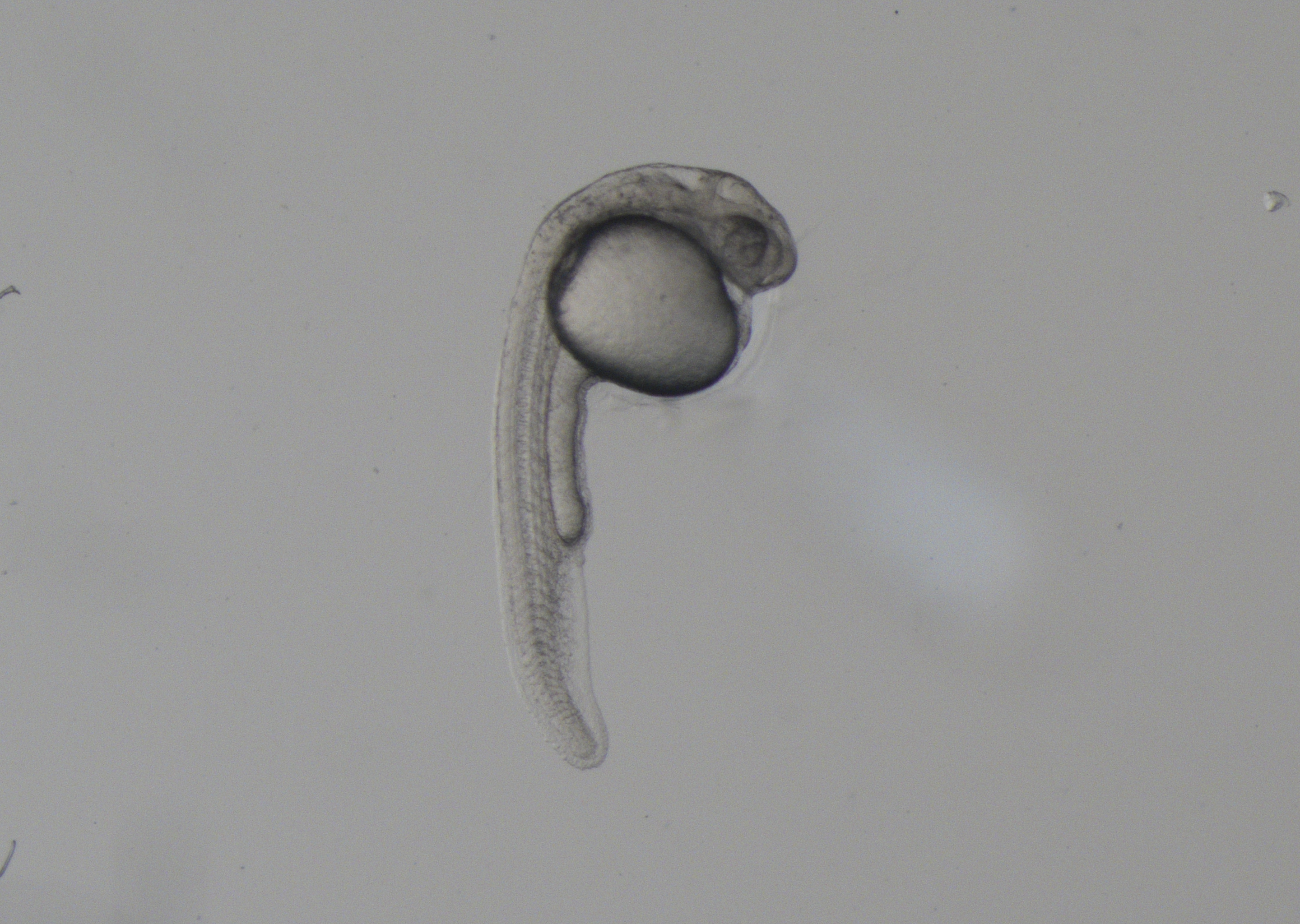

Supplement: Supplementary file 9 — EV Figure Source Data [file 44318_2025_617_MOESM9_ESM.zip › Images_EV1F/ClassI.tif]

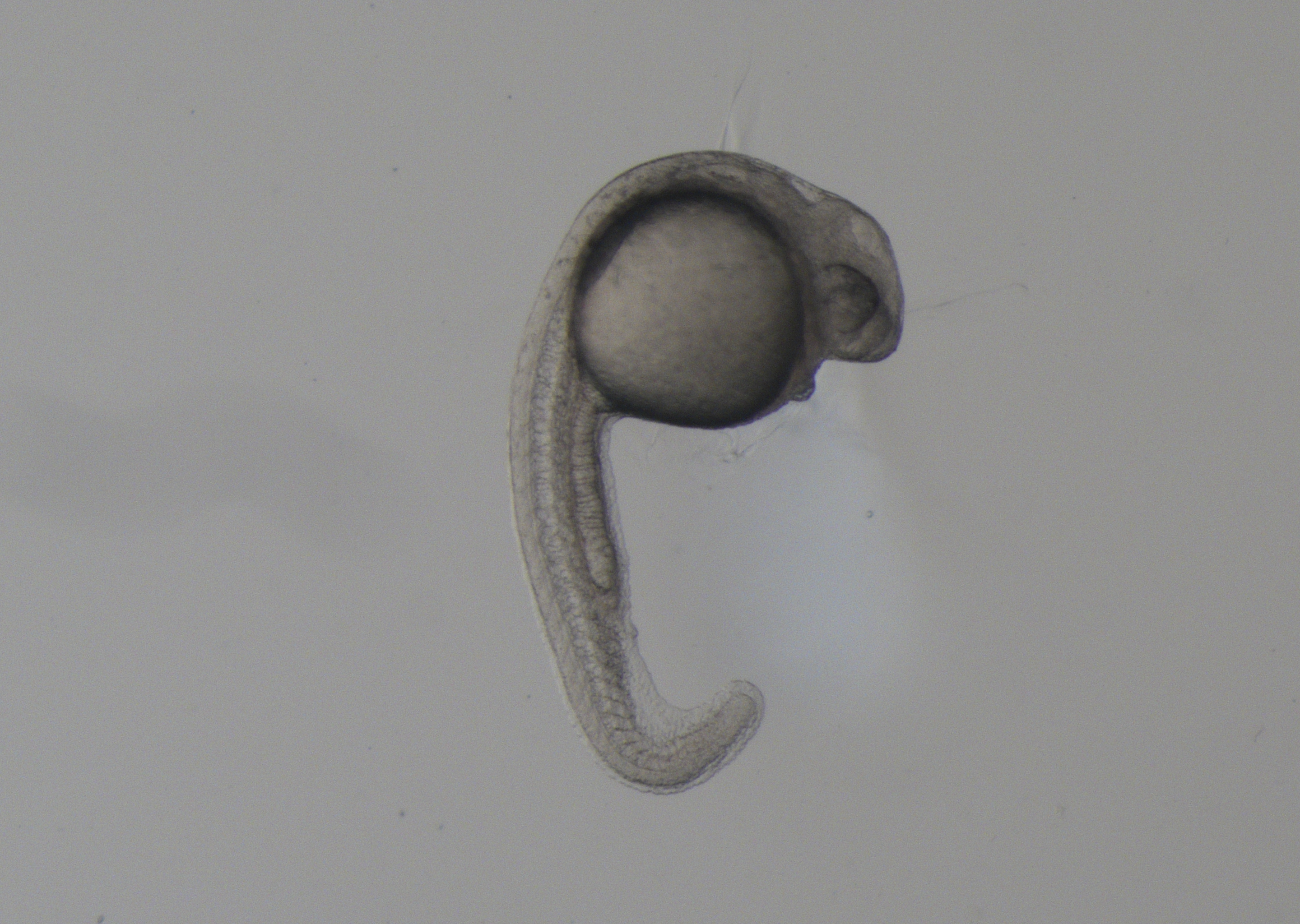

Supplement: Supplementary file 9 — EV Figure Source Data [file 44318_2025_617_MOESM9_ESM.zip › Images_EV1F/classII.tif]

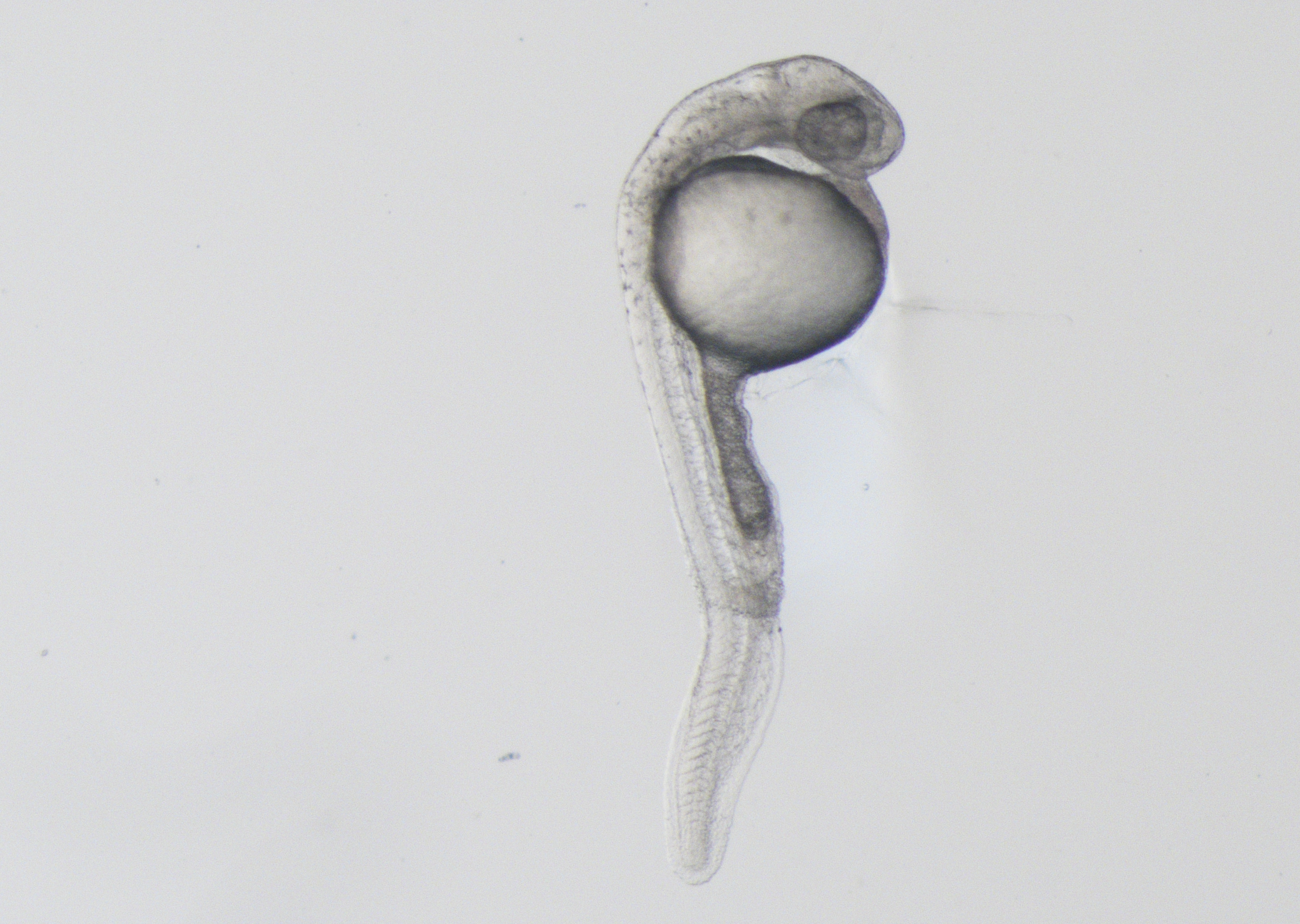

Supplement: Supplementary file 9 — EV Figure Source Data [file 44318_2025_617_MOESM9_ESM.zip › Images_EV1F/classIII.tif]

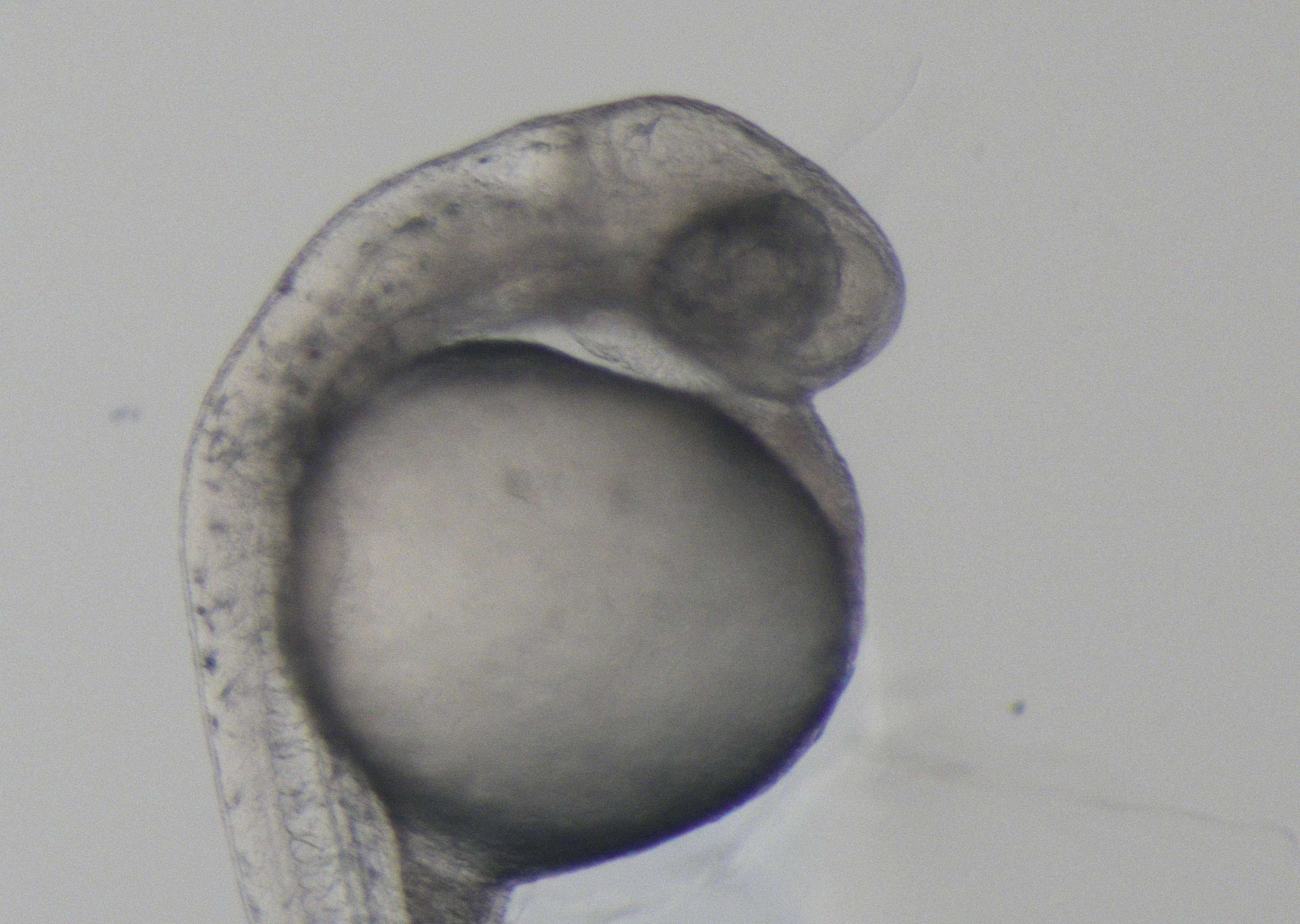

Supplement: Supplementary file 9 — EV Figure Source Data [file 44318_2025_617_MOESM9_ESM.zip › Images_EV1F/classIII_zoom.tif]

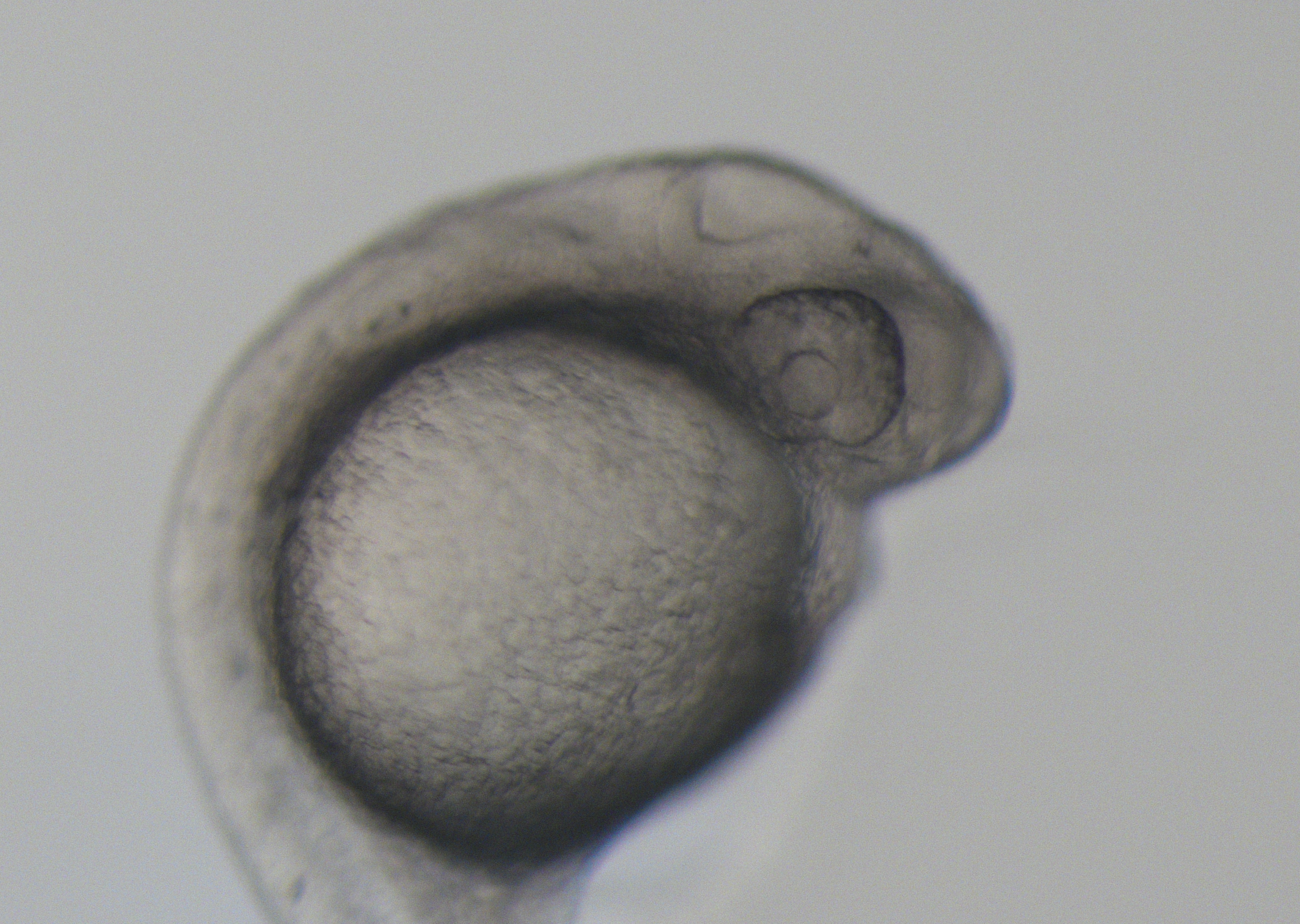

Supplement: Supplementary file 9 — EV Figure Source Data [file 44318_2025_617_MOESM9_ESM.zip › Images_EV1F/classII_zoom.tif]

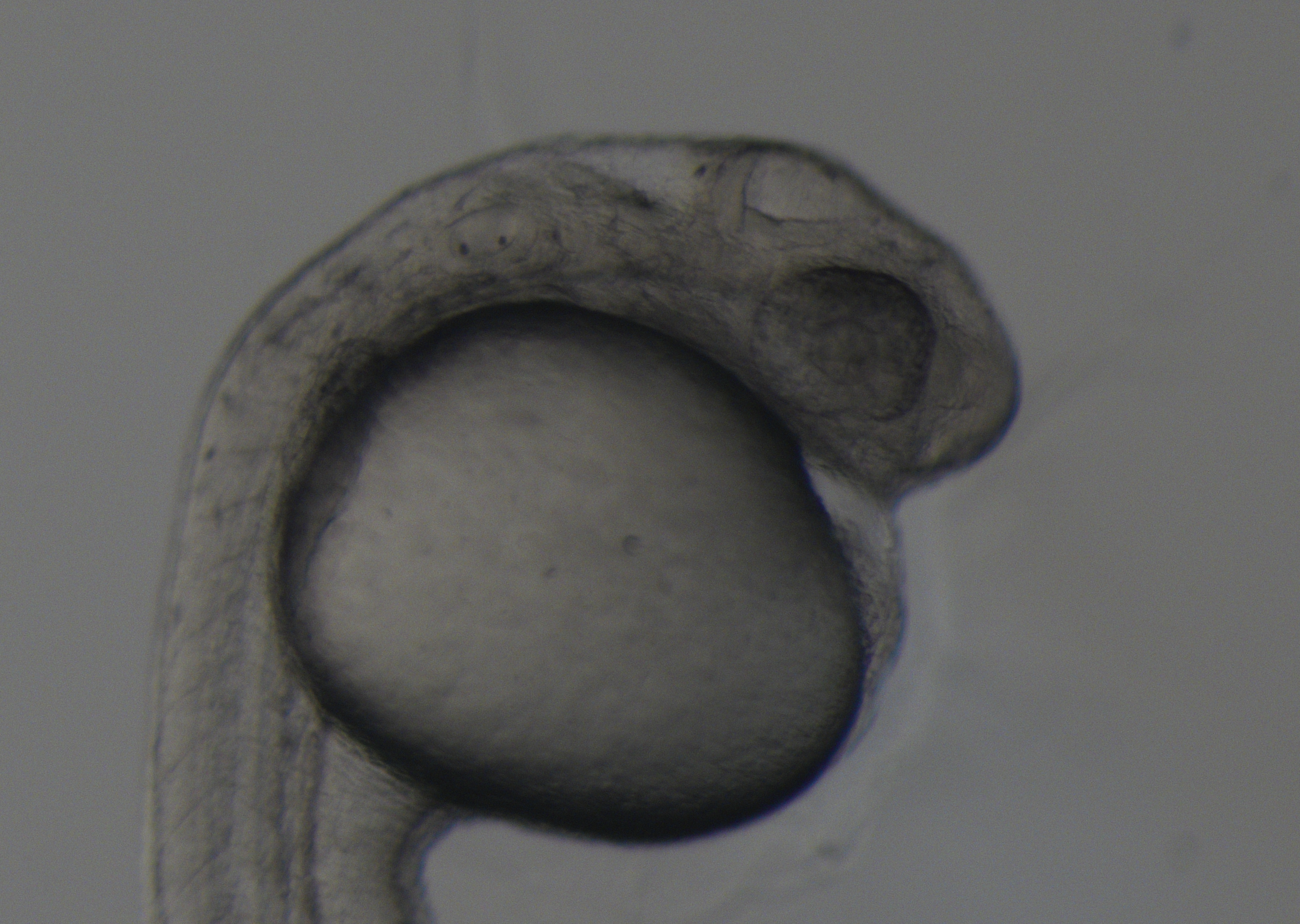

Supplement: Supplementary file 9 — EV Figure Source Data [file 44318_2025_617_MOESM9_ESM.zip › Images_EV1F/classI_zoom.tif]

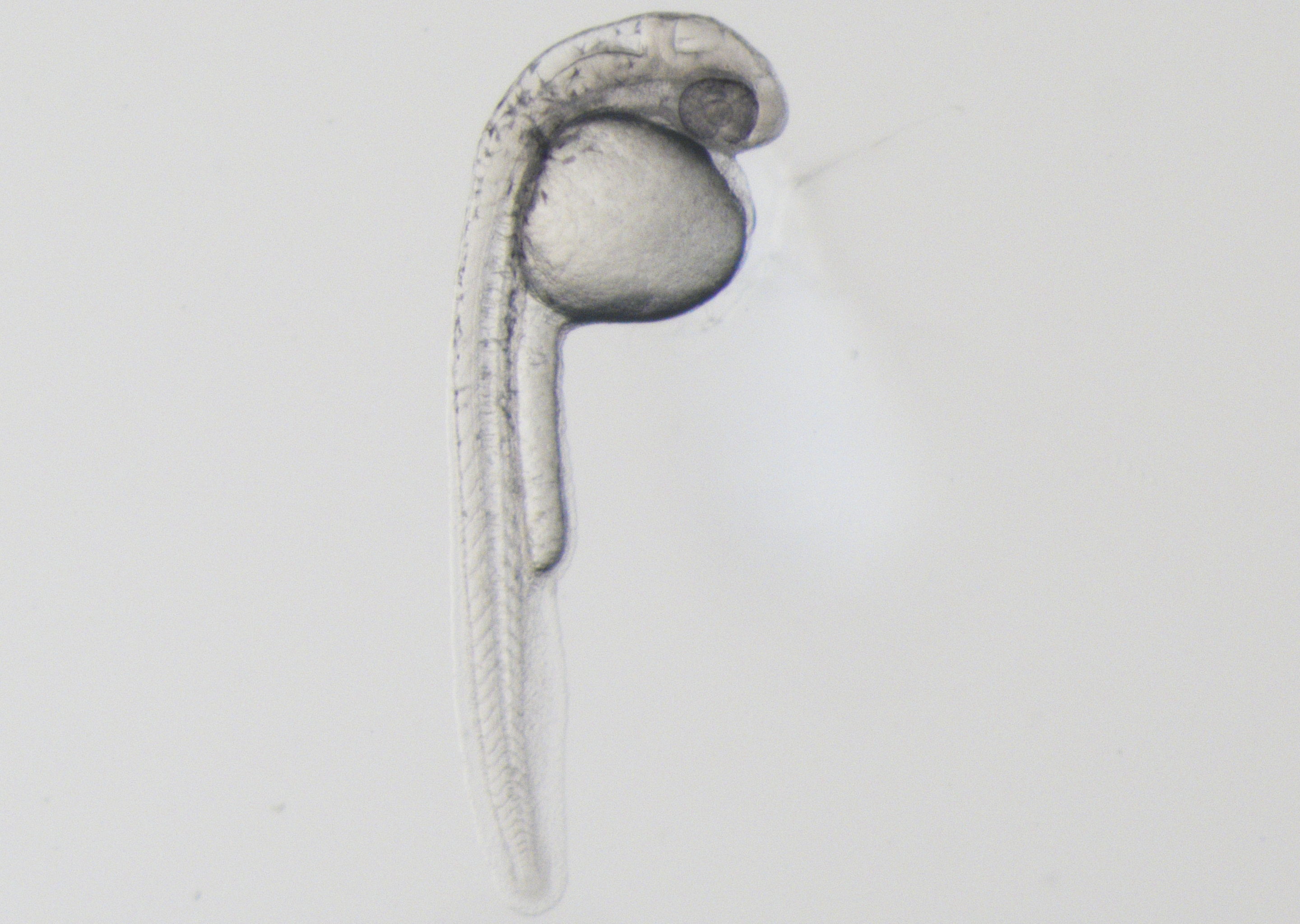

Supplement: Supplementary file 9 — EV Figure Source Data [file 44318_2025_617_MOESM9_ESM.zip › Images_EV1F/WT.tif]

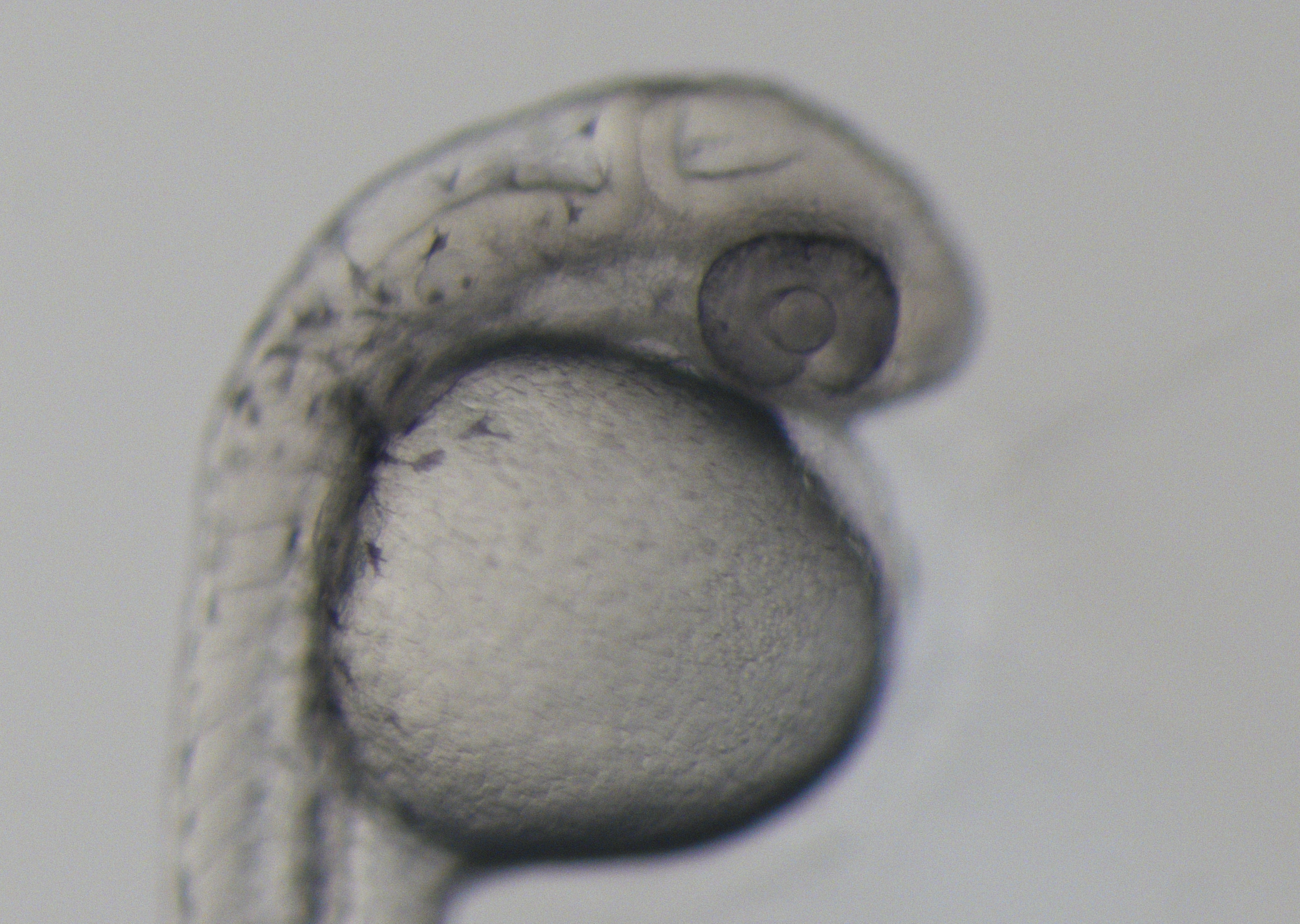

Supplement: Supplementary file 9 — EV Figure Source Data [file 44318_2025_617_MOESM9_ESM.zip › Images_EV1F/WT_zoom.tif]

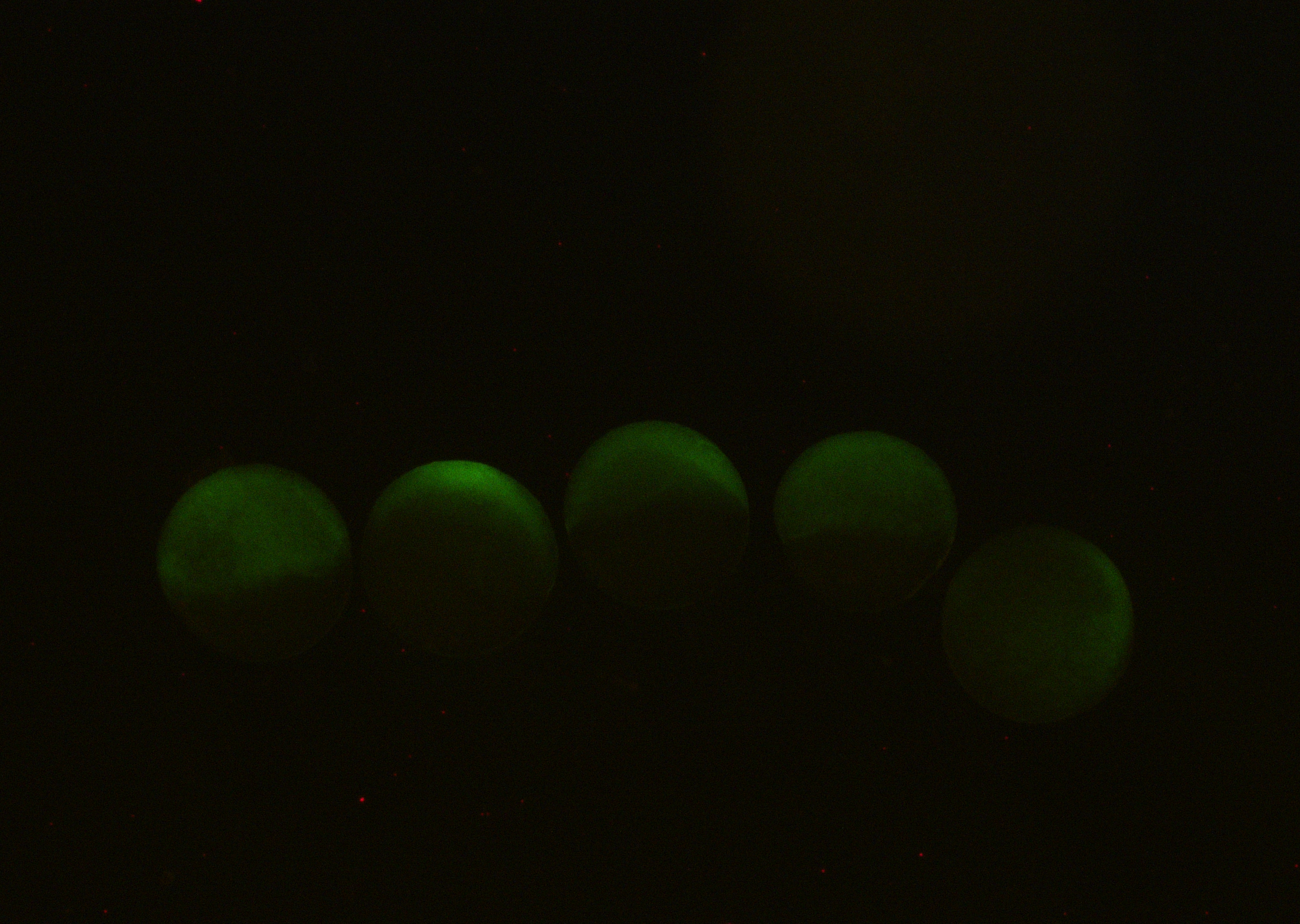

Supplement: Supplementary file 9 — EV Figure Source Data [file 44318_2025_617_MOESM9_ESM.zip › Images_EV5G/bckdk_KD_GFP.tif]

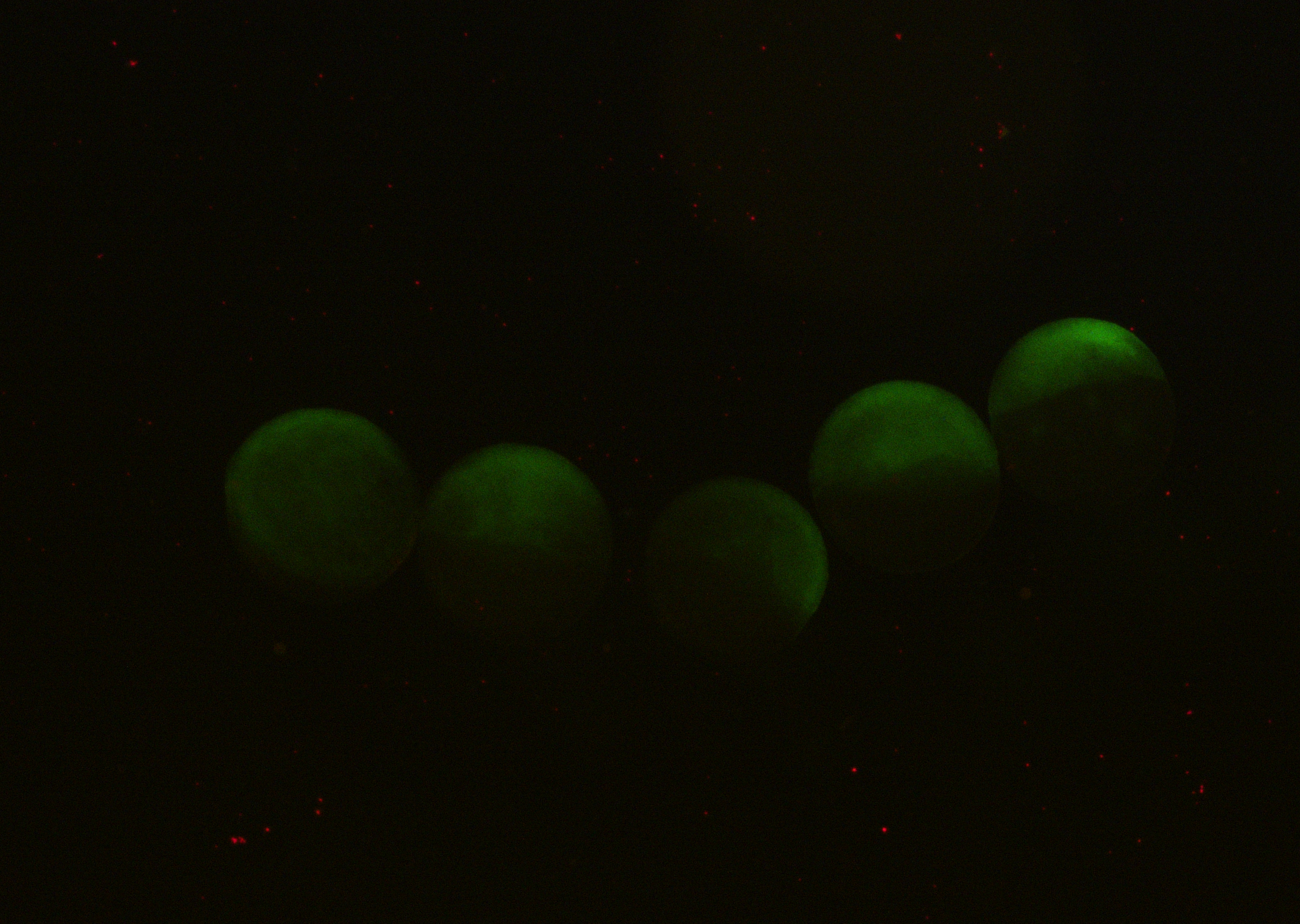

Supplement: Supplementary file 9 — EV Figure Source Data [file 44318_2025_617_MOESM9_ESM.zip › Images_EV5G/Bckdk_KD_UTR_GFP.tif]

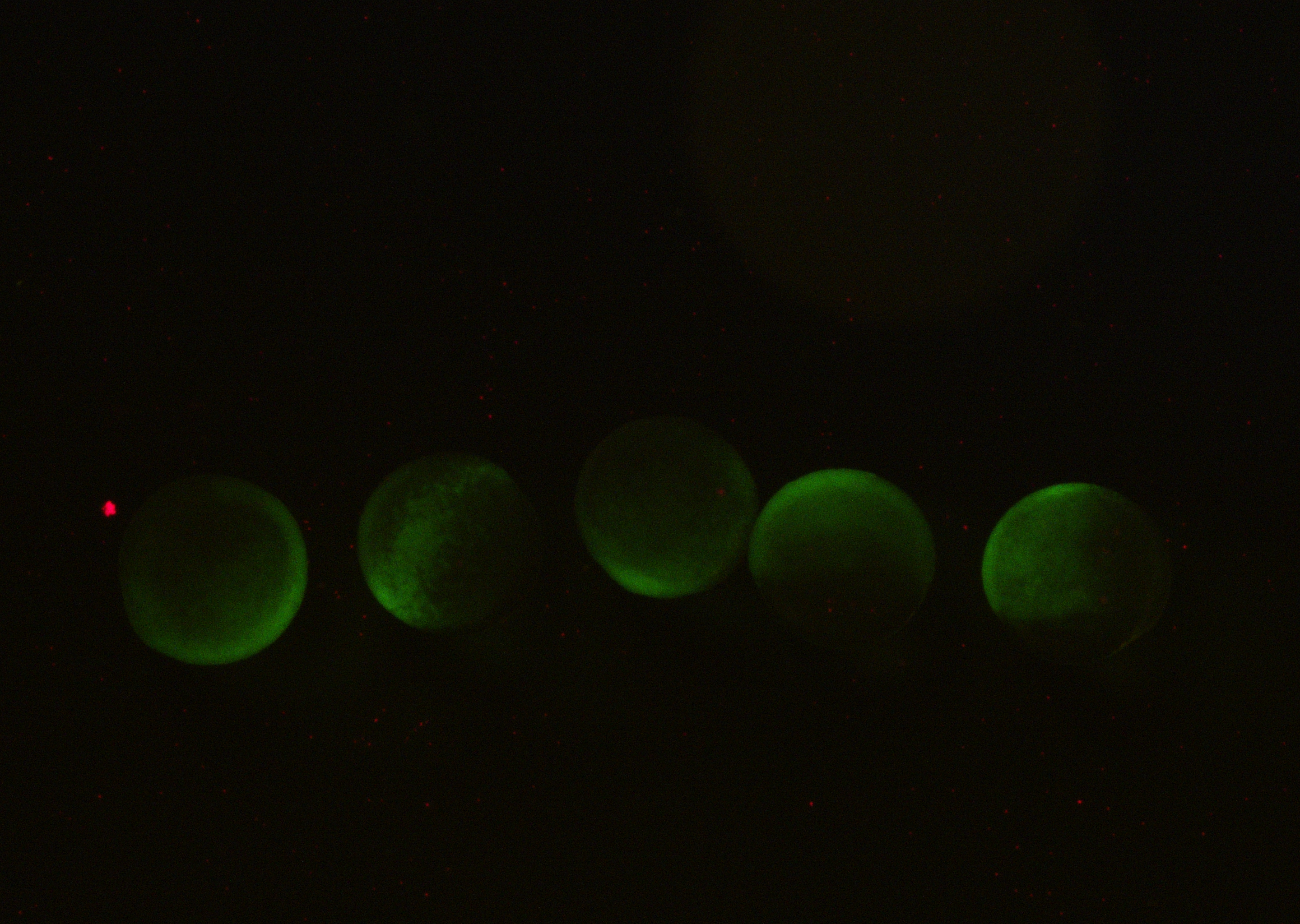

Supplement: Supplementary file 9 — EV Figure Source Data [file 44318_2025_617_MOESM9_ESM.zip › Images_EV5G/Cas13d_GFP.tif]
