# Supplementary material for: CRISPR-RfxCas13d screening uncovers Bckdk as a post-translational regulator of maternal-to-zygotic transition in teleosts
Source: EMBO J. 2025 Nov 18;44(23):7021–59. doi: 10.1038/s44318-025-00617-8 (PMC12669676; doi:10.1038/s44318-025-00617-8)
Supplement: Supplementary file 10 — Expanded View Figures [file 44318_2025_617_MOESM10_ESM.pdf]

Expanded View Figures

Figure EV1. CRISPR-RfxCas13d maternal screening identifies candidates with a role along and after MZT in zebrafish.

(**A**) RT-qPCR analysis showing levels of *nanog* mRNA at 4 hpf in the indicated conditions. Results are shown as the averages ± standard error of the mean from two experiments with two biological replicates per experiment ($n = 10$ embryos/biological replicate) for RfxCas13d protein alone (Cas13d) and RfxCas13d plus a mix of three gRNAs targeting *nanog* mRNA (Nanog KD). *taf15* mRNA was used as a normalization control. Exact *p* value is indicated above, an unpaired *t*-test. (**B**) Stacked barplots showing the percentage of wildtype (WT), developmentally altered or dead zebrafish embryos at 24 hpf after the depletion of *bckdk*, *cab39l*, *calm1a*, *calm2a*, *mknk1*, *mknk2a*, and *ppp4r2a* mRNAs. Embryos were previously divided at 6 hpf according to their developmental phenotype. No: embryos between germ ring and shield stage at 6 hpf. Yes: embryos between 30 and 50% epiboly at 6 hpf. The results are shown as the averages ± standard error of the mean of each developmental stage from two independent experiments. mRNAs encoding for kinases are labeled in blue and mRNAs encoding for phosphatases are labeled in green. The number of embryos evaluated (*n*) for each condition is shown. (**C**) Stacked barplots showing the percentage of wildtype and developmentally altered embryos from the CRISPR-RfxCas13d screening conditions that did not present more than 35% of embryos with epiboly defects at 6 hpf (Fig. 1C). The results are shown as the averages ± standard error of the mean of each developmental stage from at least two independent experiments. mRNAs encoding for kinases are labeled in blue and mRNAs encoding for phosphatases are labeled in green. The number of embryos evaluated (*n*) for each condition is shown. (**D**) Representative pictures of embryos injected with RfxCas13d and three gRNAs targeting *mibp* mRNA (gMIBP) compared to uninjected embryos (WT) evaluated at 30 hpf (scale bar, 0.5 mm). Class I: curved tail (mild phenotype). Class II: shorter tail and partial microcephaly (severe phenotype). Class III: notochord malformation and microcephaly (extremely severe phenotype). (**E**) Stacked barplots showing percentage of observed phenotypes at 30 hpf after the injection of RfxCas13d protein (Cas13d) (3 ng/embryo) alone or together with a mix of 3 gRNAs targeting *mibp* mRNA (gMIBP) (1000 pg/embryo). The results are shown as the averages ± standard error of the mean of each developmental stage from three independent experiments. The phenotype selection follows the criteria of panel (**D**). The number of embryos evaluated (*n*) for each condition is shown. (**F**) RT-qPCR analysis showing levels of *mibp* mRNA at 4 hpf in the indicated conditions. Results are shown as the averages ± standard error of the mean from two experiments with two biological replicates per experiment ($n = 10$ embryos/biological replicate) for RfxCas13d protein alone (Cas13d) and RfxCas13d plus a mix of 3 gRNAs targeting *mibp* mRNA (gMIBP). *taf15* mRNA was used as a normalization control. Exact *p* value is indicated above (unpaired *t*-test). (**G**) Intersection visualization analysis showing the number of downregulated genes shared between the mRNA knockdowns. Intersections with more than 50 shared genes are shown. Source data are available online for this figure.

© The Author(s)

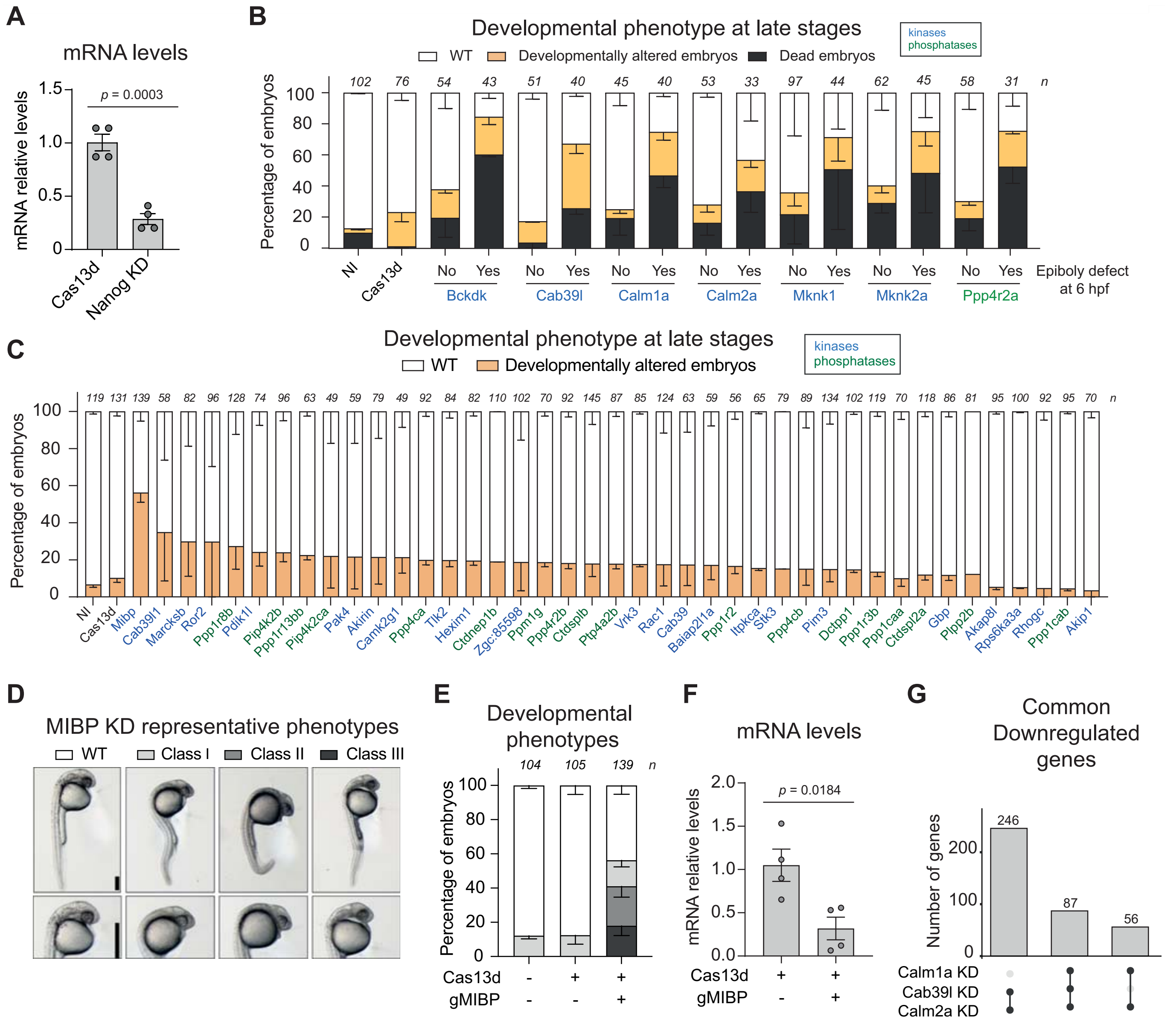
A
mRNA levels
mRNA relative levels
p = 0.0003
Cas13d
Nanog KD
B
Developmental phenotype at late stages
kinases
phosphatases
WT
Developmentally altered embryos
Dead embryos
Percentage of embryos
102 76 54 43 51 40 45 40 53 33 97 44 62 45 58 31 n
NI
Cas13d
No Yes
Bckdk
Cab39l
Calm1a
Calm2a
Mknk1
Mknk2a
Ppp4r2a
Epiboly defect at 6 hpf
C
Developmental phenotype at late stages
kinases
phosphatases
WT
Developmentally altered embryos
Percentage of embryos
119 131 139 58 82 96 128 74 96 63 49 59 79 49 92 84 82 110 102 70 92 145 87 85 124 63 59 56 65 79 89 134 102 119 70 118 86 81 95 100 92 95 70 n
NI
Cas13d
Mibp
Cab39l1
Marcksb
Ror2
Ppp1r8b
Pdik1l
Pip4k2b
Ppp1r13bb
Pip4k2ca
Pak4
Akirin
Camk2g1
Ppp4ca
Tlk2
Hexim1
Ctdnep1b
Zgc:85598
Ppm1g
Ppp4r2b
Ctdsplb
Ptp4a2b
Vrk3
Rac1
Cab39
Baiap2l1a
Ppp1r2
Itpkca
Stk3
Ppp4cb
Pim3
Dctpp1
Ppp1r3b
Ppp1caa
Ctdspl2a
Gbp
Plpp2b
Akap8l
Rps6ka3a
Rhogc
Ppp1cab
Akip1
D
MIBP KD representative phenotypes
WT
Class I
Class II
Class III
E
Developmental phenotypes
104 105 139 n
Percentage of embryos
Cas13d
gMIBP
F
mRNA levels
mRNA relative levels
p = 0.0184
Cas13d
gMIBP
G
Common Downregulated genes
Number of genes
246
87
56
Calm1a KD
Cab39l KD
Calm2a KD

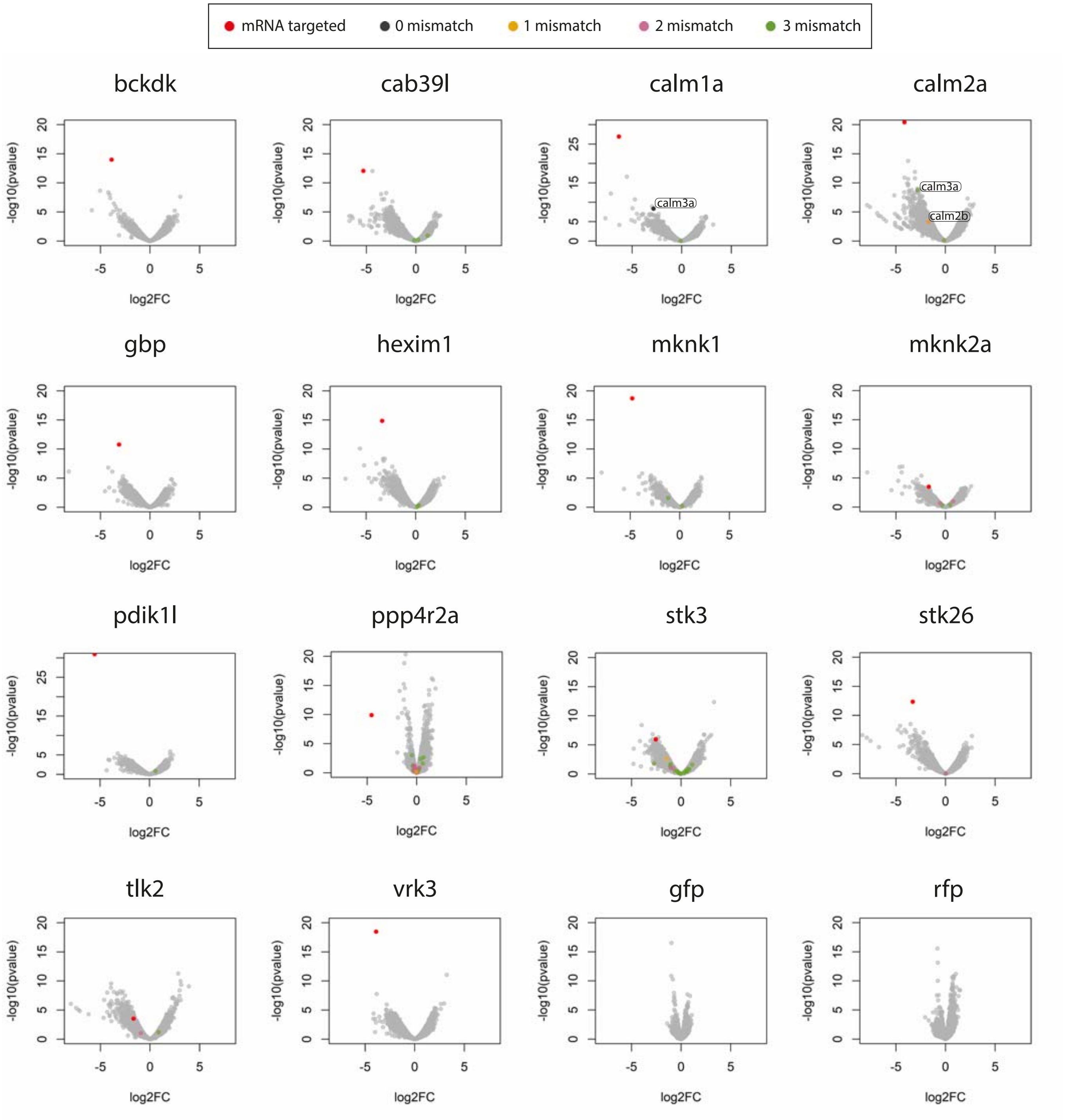

Figure EV2. CRISPR-RfxCas13d triggers efficient and specific depletion of targeted mRNAs with minimal off-target effects.

Scatter plots showing the fold change in mRNA levels and *p* values from a minimum of two biological RNA-seq replicates at 4 hpf. *p* values were calculated using the Wald test. Data are shown for seven candidates that caused epiboly defects (positive candidates) in at least 35% of injected embryos, seven candidates that did not meet this developmental phenotype threshold (negative candidates), and two non-targeting control conditions (gRNAs designed to target *gfp* and *rfp* mRNA). The mRNA targeted in each condition is highlighted in red. mRNAs that could potentially be recognized by the gRNAs used in each condition (allowing up to three mismatches) are also indicated: zero mismatches in black, one mismatch in yellow, two mismatches in pink, and three mismatches in green.

 © The Author(s)

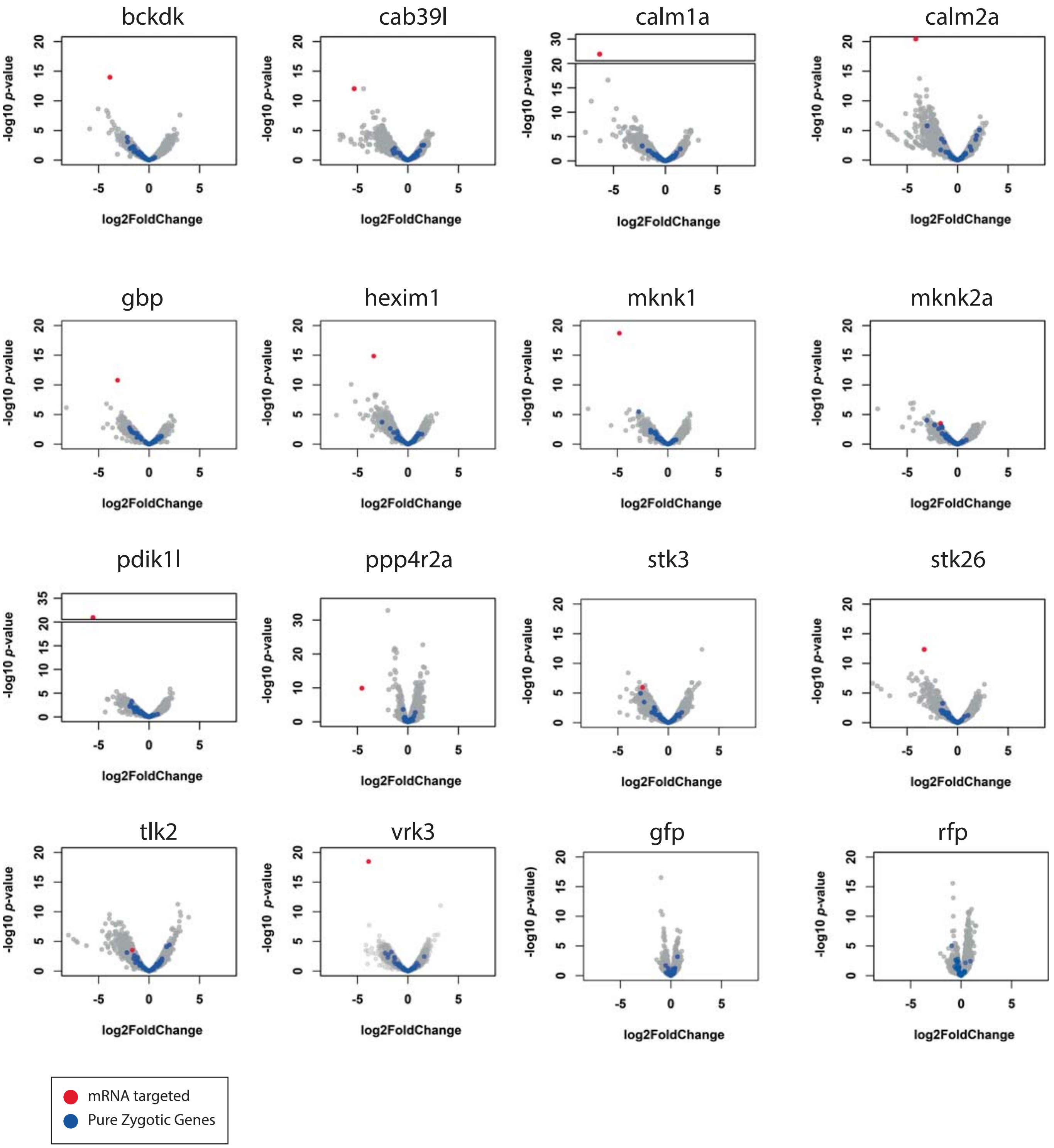

Figure EV3. Transcriptome analysis shows a global downregulation of PZG upon *bckdk* and *mknk2a* mRNA depletion.

Scatter plots representing the fold change in mRNA levels and *p* value from a minimum of two biological RNA-seq replicates at 4 hpf of seven candidates with epiboly defects (positive candidate) in at least 35% of injected embryos and seven candidates that did not pass this developmental phenotype filter (negative candidate) and two non-targeting control conditions (gRNAs designed to target *gfp* and *rfp* mRNA). *p* values were calculated using the Wald test. Pure zygotic genes mRNAs defined by Lee et al, 2013 are indicated in blue. The mRNA targeted in each condition is highlighted in red.

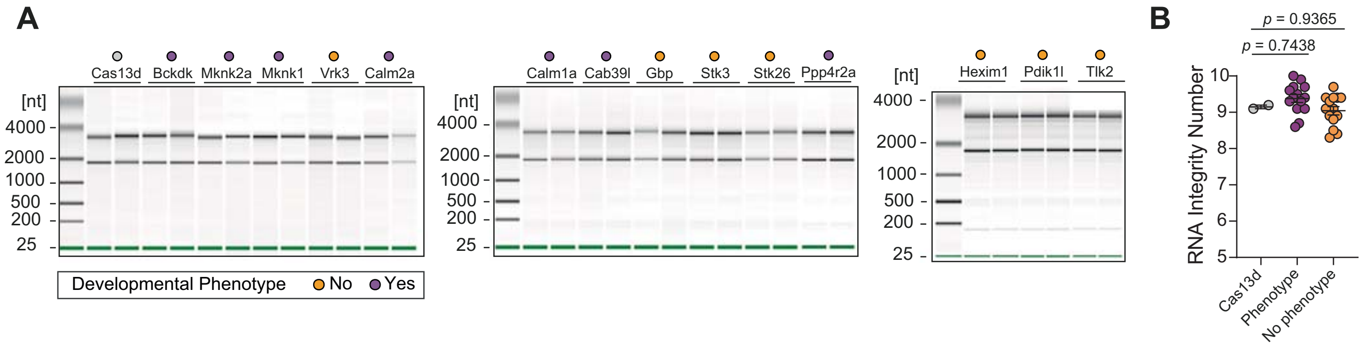

Figure EV4. RNA-targeting mediated by CRISPR-RfxCas13d RNP complexes does not show collateral activity in zebrafish embryos.

RNA integrity analysis (RIN) from samples used for RNA-Seq in Figs. EV2 and EV3 analysed by Agilent Bioanalyzer 2100. Electrophoresis gel (**A**) and RIN-associated (**B**). Developmental phenotype associated to each KD condition is indicated as purple dots (positive candidate) or orange dots (negative candidate). The RfxCas13d control condition is indicated as gray dots. One-way ANOVA comparing RIN from all the samples between them is shown in (**B**). Exact *p* values are indicated above. Source data are available online for this figure.

 © The Author(s)

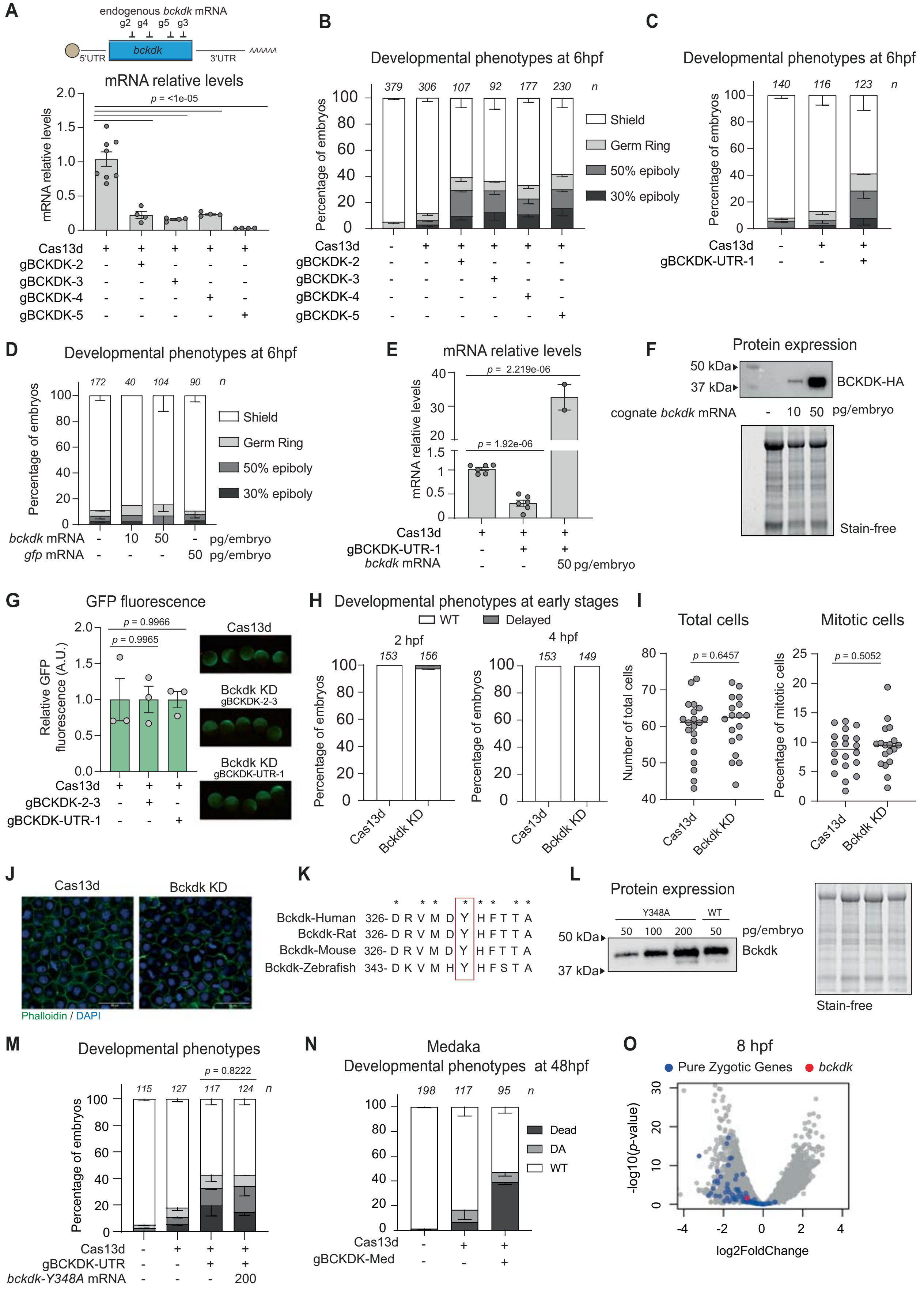
A
endogenous bckdk mRNA
g2 g4 g5 g3
bckdk
5'UTR
3'UTR
AAAAAA
mRNA relative levels
p = <1e-05
mRNA relative levels
Cas13d
gBCKDK-2
gBCKDK-3
gBCKDK-4
gBCKDK-5
B
Developmental phenotypes at 6hpf
379 306 107 92 177 230 n
Shield
Germ Ring
50% epiboly
30% epiboly
Percentage of embryos
C
Developmental phenotypes at 6hpf
140 116 123 n
gBCKDK-UTR-1
D
Developmental phenotypes at 6hpf
172 40 104 90 n
bckdk mRNA - 10 50 - pg/embryo
gfp mRNA - - - 50 pg/embryo
E
mRNA relative levels
p = 2.219e-06
p = 1.92e-06
bckdk mRNA - - 50 pg/embryo
F
Protein expression
50 kDa
37 kDa
BCKDK-HA
cognate bckdk mRNA - 10 50 pg/embryo
Stain-free
G
GFP fluorescence
p = 0.9966
p = 0.9965
Relative GFP fluorescence (A.U.)
Cas13d
Bckdk KD gBCKDK-2-3
Bckdk KD gBCKDK-UTR-1
gBCKDK-2-3
gBCKDK-UTR-1
H
Developmental phenotypes at early stages
WT
Delayed
2 hpf
4 hpf
153 156
153 149
Cas13d
Bckdk KD
I
Total cells
Mitotic cells
p = 0.6457
p = 0.5052
Number of total cells
Pecentage of mitotic cells
J
Cas13d
Bckdk KD
Phalloidin / DAPI
K
Bckdk-Human 326- D R V M D Y H F T T A
Bckdk-Rat 326- D R V M D Y H F T T A
Bckdk-Mouse 326- D R V M D Y H F T T A
Bckdk-Zebrafish 343- D K V M H Y H F S T A
L
Protein expression
Y348A
WT
50 100 200 50 pg/embryo
Bckdk
50 kDa
37 kDa
Stain-free
M
Developmental phenotypes
p = 0.8222
115 127 117 124 n
gBCKDK-UTR
bckdk-Y348A mRNA - - - 200
N
Medaka
Developmental phenotypes at 48hpf
198 117 95 n
Dead
DA
WT
gBCKDK-Med
O
8 hpf
Pure Zygotic Genes
bckdk
-log10(p-value)
log2FoldChange

Figure EV5. The developmental phenotype upon *bckdk* mRNA depletion can be genetically rescued and is recapitulated using independent gRNAs and in different teleost models.

(**A**) Schematic representation of endogenous *bckdk* mRNA and individual gRNAs employed (Top). RT-qPCR analysis showing levels of *bckdk* mRNA at 4 hpf in zebrafish embryos co-injected using individual gRNAs targeting *bckdk* mRNA and RfxCas13d protein. Results are shown as the averages ± standard error of the mean from two independent experiments with four biological replicates each ($n = 10$ embryos/biological replicate). *taf15* mRNA was used as a normalization control. Exact *p* values are indicated above (one-way ANOVA) (Bottom). (**B**) Stacked barplots showing the percentage of zebrafish embryos in different developmental stages quantified at 6 hpf using individual gRNAs (1000 pg/embryo) targeting *bckdk* mRNA co-injected with RfxCas13d protein (3 ng/embryo). The phenotype selection criteria were the same as described in Fig. 1C. The results are shown as the averages ± standard error of the mean of each developmental stage from at least two independent experiments. The number of embryos evaluated (*n*) is shown for each condition. (**C**) Stacked barplots showing developmental phenotypes at 6 hpf upon depletion of *bckdk* mRNA using a gRNA targeting the 3′UTR (gBCKDK-UTR-1). The phenotype selection criteria were the same as described in Fig. 1C. The results are shown as the averages ± standard error of the mean of each developmental stage from two independent experiments. The number of embryos evaluated (*n*) is shown for each condition. (**D**) Stacked barplots showing developmental phenotypes at 6 hpf in zebrafish embryos injected with the cognate *bckdk* mRNA at 10 or 50 pg per embryo or with an mRNA encoding for GFP at 50 pg per embryo. The phenotype selection criteria were the same as described in Fig. 1C. The results are shown as the averages ± standard error of the mean of each developmental stage from two independent experiments. The number of embryos evaluated (*n*) is shown for each condition. (**E**) RT-qPCR analysis showing levels of *bckdk* mRNA at 4 hpf in zebrafish embryos in the rescue experiment (Fig. 2B). Results are shown as the averages ± standard error of the mean from one to three independent experiments with at least two biological replicates each ($n = 10$ embryos/ biological replicate). *taf15* mRNA was used as a normalization control. Exact *p* values are indicated above (unpaired *t*-test). (**F**) Western blot showing Bckdk-HA protein expression at 6 hpf in zebrafish embryos injected with 10 or 50 pg/embryo of the cognate *bckdk-HA* mRNA (Top panel). Bottom panel shows stain-free signal (Gürtler et al, 2013) of the gel as loading control. (**G**) Barplots showing GFP fluorescence signal in zebrafish embryos injected with RfxCas13d alone (Cas13d) or with 2 gRNAs targeting *bckdk* mRNA in the coding sequence (Bckdk KD; gBCKDK-2-3) or one in the 3′UTR (Bckdk KD; gBCKDK-UTR-1) together with 50 pg of *gfp* mRNA. GFP signal is quantified from three biological replicates of five embryos each. Exact *p* values are indicated above, unpaired *t*-test). Representative fluorescence microscopy images used for the quantification are shown. (**H**) Stacked barplots representing the percentage of embryos normally developed (WT) or delayed at 2 or 4 hpf in zebrafish embryos injected with RfxCas13d protein alone (Cas13d) (3 ng/embryo) or with a mix of three gRNAs (1000 pg/embryo) targeting *bckdk* mRNA (Bckdk KD). The results are shown as the averages ± standard error of the mean of each developmental stage from two independent experiments. Number of embryos evaluated (*n*) is shown for each condition. (**I**) Number of total cells or percentage of mitotic cells in zebrafish embryos at 4 hpf injected with RfxCas13d protein alone (Cas13d) or with a mix of three gRNAs targeting *bckdk* mRNA (Bckdk KD). The black line represents the median from two independent experiments with at least four embryos each. Exact *p* values are indicated above (unpaired *t*-test). (**J**) Representative immunofluorescence used for quantification in panel (**I**) showing zebrafish embryos injected with RfxCas13d alone (Cas13d) or with gRNAs targeting *bckdk* mRNA (Bckdk KD), fixed at 4 hpf with PFA 4% and incubated with Phalloidin and DAPI (see Methods for details; Scale bar, 100 μm). (**K**) Schematic representation of the Bckdk protein alignment among human, mouse, rat, and zebrafish. The red square highlights a conserved tyrosine residue, whose substitution with alanine has been reported to reduce kinase activity by 95% (Wynn et al, 2000; Singh et al, 2024). Asterisks (*) indicate amino acids conserved across all analyzed species. (**L**) Western blot showing Bckdk-HA protein expression at 6 hpf in zebrafish embryos injected with 50, 100, or 200 pg/embryo of the kinase-dead *bckdk* mRNA variant, or with 50 pg of the wild-type bckdk mRNA (left panel). The right panel shows the Stain-Free gel signal used as a loading control (Gürtler et al, 2013). (**M**) Stacked barplots showing the percentage of observed phenotypes in zebrafish embryos injected with RfxCas13d protein (3 ng/embryo) together with one gRNA targeting the 3′UTR of the endogenous *bckdk* mRNA (gBCKDK-UTR) (1000 pg/embryo), and co-injected with a dead-kinase *bckdk* mRNA version (200 pg/embryo). The results are shown as the averages ± standard error of the mean of each developmental stage from two independent experiments. The number of embryos evaluated (*n*) is shown for each condition. Exact *p* value is indicated above ($\chi 2$-test). (**N**) Stacked barplots representing the percentage at 48 hpf of wildtype (WT), developmentally altered (DA) or dead medaka embryos injected with RfxCas13d protein (6 ng/embryo) alone or together with a gRNA (gBCKDK-Med) (2000 pg/embryo) targeting *bckdk* mRNA. The results are shown as the averages ± standard error of the mean of each developmental stage from three independent experiments. The number of embryos evaluated (*n*) is shown for each condition. (**O**) Scatter plots representing the log2 fold change in mRNA level and the associated *p* value from three biological RNA-seq replicates ($n = 10$ embryos/biological replicate) at 8 hpf in medaka embryos from the comparison between embryos injected only with RfxCas13d protein or together with a gRNA targeting *bckdk* mRNA. *bckdk* mRNA is represented in red. Pure zygotic genes mRNAs (PZG) from medaka embryos determined by Li et al, 2020 data were depicted in blue. Source data are available online for this figure.

 © The Author(s)

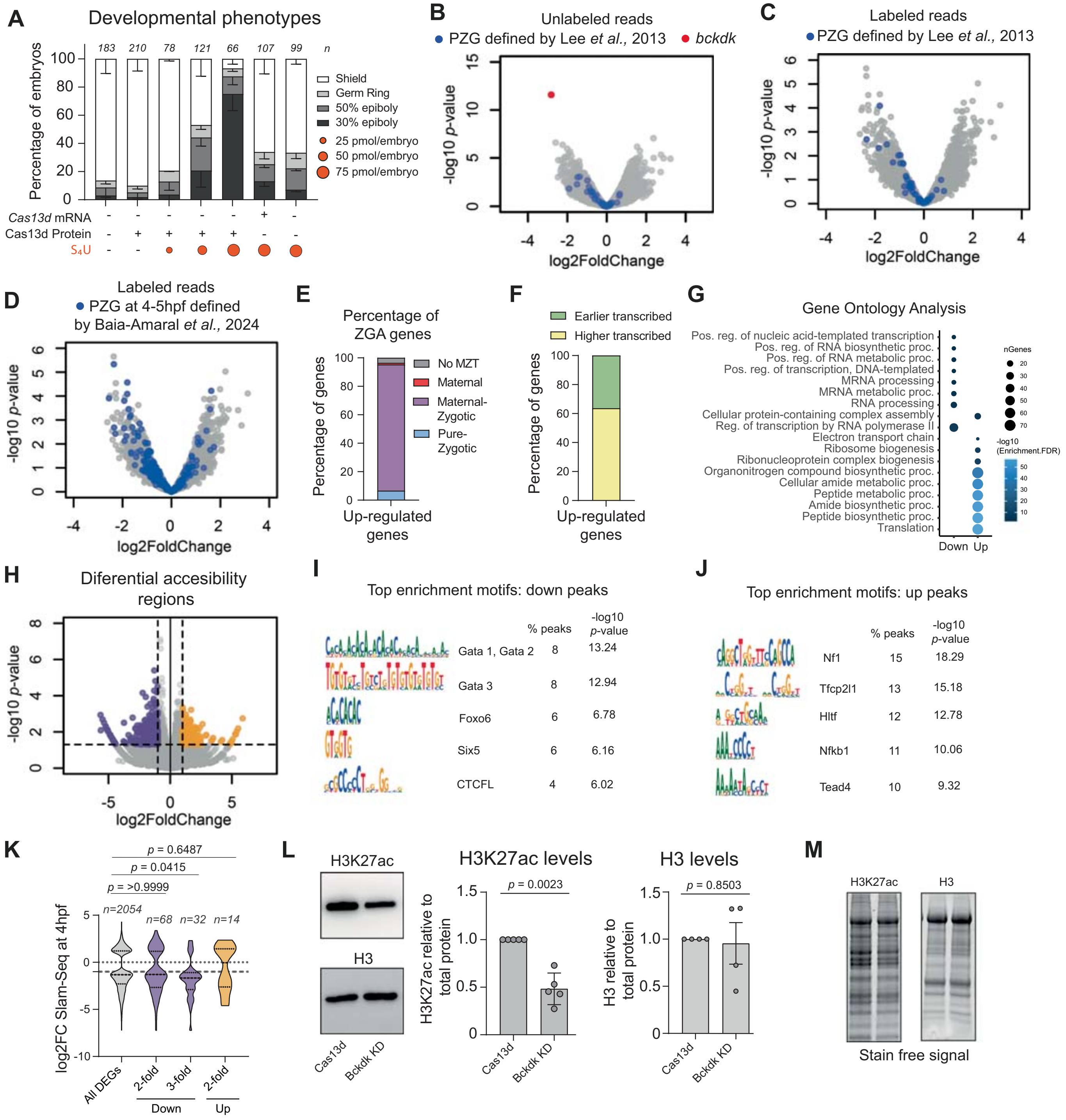

A
Developmental phenotypes
Percentage of embryos
183 210 78 121 66 107 99 n
Shield
Germ Ring
50% epiboly
30% epiboly
25 pmol/embryo
50 pmol/embryo
75 pmol/embryo
Cas13d mRNA
Cas13d Protein
S4U
B
Unlabeled reads
PZG defined by Lee et al., 2013
bckdk
-log10 p-value
log2FoldChange
C
Labeled reads
PZG defined by Lee et al., 2013
D
Labeled reads
PZG at 4-5hpf defined by Baia-Amaral et al., 2024
E
Percentage of ZGA genes
Percentage of genes
No MZT
Maternal
Maternal-Zygotic
Pure-Zygotic
Up-regulated genes
F
Earlier transcribed
Higher transcribed
G
Gene Ontology Analysis
Pos. reg. of nucleic acid-templated transcription
Pos. reg. of RNA biosynthetic proc.
Pos. reg. of RNA metabolic proc.
Pos. reg. of transcription, DNA-templated
MRNA processing
MRNA metabolic proc.
RNA processing
Cellular protein-containing complex assembly
Reg. of transcription by RNA polymerase II
Electron transport chain
Ribosome biogenesis
Ribonucleoprotein complex biogenesis
Organonitrogen compound biosynthetic proc.
Cellular amide metabolic proc.
Peptide metabolic proc.
Amide biosynthetic proc.
Peptide biosynthetic proc.
Translation
Down Up
nGenes
-log10 (Enrichment.FDR)
H
Diferential accesibility regions
I
Top enrichment motifs: down peaks
% peaks
-log10 p-value
Gata 1, Gata 2 8 13.24
Gata 3 8 12.94
Foxo6 6 6.78
Six5 6 6.16
CTCFL 4 6.02
J
Top enrichment motifs: up peaks
Nf1 15 18.29
Tfcp2l1 13 15.18
Hltf 12 12.78
Nfkb1 11 10.06
Tead4 10 9.32
K
p = 0.6487
p = 0.0415
p = >0.9999
n=2054 n=68 n=32 n=14
log2FC Slam-Seq at 4hpf
All DEGs 2-fold 3-fold 2-fold
Down Up
L
H3K27ac
H3
Cas13d Bckdk KD
H3K27ac levels
p = 0.0023
H3K27ac relative to total protein
H3 levels
p = 0.8503
H3 relative to total protein
M
H3K27ac H3
Stain free signal

Figure EV6. SLAM-seq and ATAC analysis in *bckdk* mRNA KD.

(**A**) Stacked barplots showing developmental phenotypes at 6 hpf in zebrafish embryos injected with RfxCas13d protein (3 ng/embryo) or *RfxCas13d* mRNA (150 pg/embryo) alone or together with different amounts of S_4U (25, 50, or 75 mM). The phenotype selection criteria were the same as described in Fig. 1C. The results are shown as the averages ± standard error of the mean of each developmental stage from at least two independent experiments. The number of embryos evaluated (*n*) is shown for each condition. (**B**) Volcano plot representing the log2 fold change in mRNA level from unlabeled reads (SLAM-Seq data) and the associated *p* value from two and four biological replicates (*n* = 25 embryos/biological replicate) at 4 hpf from embryos injected with RfxCas13d protein alone or with a mix of two gRNAs targeting *bckdk* mRNA, respectively. *p* values were calculated using the Wald test. *bckdk* mRNA is represented in red. Pure zygotic genes mRNAs (PZG) determined by Lee et al, 2013 data were depicted in blue. Volcano plots representing the fold change in mRNA level from labeled reads and the associated *p* value from three biological SLAM-Seq replicates of zebrafish embryos at 4 hpf from two and four biological replicates (*n* = 25 embryos/biological replicate) from embryos injected with RfxCas13d protein alone or with a mix of two gRNAs targeting *bckdk* mRNA, respectively. *p* values were calculated using the Wald test. *bckdk* mRNA is represented in red. Pure zygotic genes mRNAs (PZG) determined by Lee et al, 2013 data were depicted in blue in panel (**C**) and an updated list of pure zygotic genes mRNAs (PZG) determined by Baia Amaral et al, 2024 data were depicted in blue in panel (**D**). (**E**) Stacked barplot showing percentage of upregulated genes in SLAM-Seq data upon *bckdk* mRNA depletion that belong to different categories. Genes were classified according to Baia Amaral et al, 2024. No MZT: Genes that are not present between 0 and 7 hpf; Maternal: Genes maternally provided as mRNA but not zygotically transcribed; Maternal-and-Zygotic: Genes maternally provided and zygotically transcribe between 4 and 7 hpf; or pure zygotic: genes not maternally provided as mRNA and zygotically transcribed between 4 and 7 hpf. (**F**) Stacked barplot showing percentage of upregulated MZT genes (maternal-and-zygotic or pure zygotic genes) in SLAM-Seq data upon *bckdk* mRNA depletion that belong to different categories. Genes were classified according to transcript levels in wild-type conditions at 4 hpf in labeled data. Higher transcribed: Genes with more than ten CPM (counts per million) in labeled data at 4 hpf in WT conditions; Earlier transcribed: Genes with less than ten CPM in labeled data at 4 hpf in WT conditions. (**G**) Gene Ontology enrichment analyses of biological processes for down-(Down) or up-regulated genes (Up) from the comparison of SLAM-Seq data between zebrafish embryos injected with RfxCas13d alone and together with two gRNAs targeting *bckdk* mRNA. Terms with a false discovery rate (FDR) lower than 0.05 and with more than 20 genes represented are shown and considered as enriched. (**H**) Differential analyses of chromatin accessibility between zebrafish embryos injected with RfxCas13d protein alone or co-injected with two gRNAs targeting *bckdk* mRNA from ATAC-seq data at 4 hpf from two biological replicates per condition (*n* = 80 embryos/biological replicate). The log2 fold change (FC) and *p* value associated is represented. *p* values were calculated using the Wald test. Regions with a significant decrease in accessibility are represented as purple dots (log2FC <-1 and *p* value <0.05), and regions with a significant increase in accessibility are represented as orange dots (log2FC >1 and *p* value <0.05). Vertical and horizontal dashed lines indicate 1.5-fold and *p* value = 0.05, respectively. Motif enrichment analyses from the decreased (**I**) and increased (**J**) ATAC regions (down and up peaks, respectively) in *bckdk* mRNA knockdown condition. The top five motifs are represented with their motif logos, transcription factor name, percentage of peaks containing the motif and enrichment *p* value. (**K**) Violin plots showing the distribution of log2 fold change of RNA levels (SLAM-Seq) from all differential expression genes (DEGs) and those associated with less accessible (Down) or more accessible (Up) regions from ATAC-Seq data. Dash lines and dot lines inside the violin plots indicate the mean and quartiles, respectively. Gray dot line and dash line outside the violin plots indicate 0-fold and 1.5-fold in RNA levels, respectively. Exact *p* values are indicated above, Mann–Whitney *U*-test. The number of differentially expressed genes (*n*) for each category are shown. (**L**) Representative western blot images for H3K27ac and H3 of embryos injected with RfxCas13d protein alone (Cas13d) or together with two gRNAs targeting bckdk mRNA (Bckdk KD) (left). Barplots represent H3K27ac or H3 levels relative to total proteins as the averages ± standard error of the mean at least four biological replicates from two or three independent experiments. Zebrafish embryos were collected at 4 hpf (*n* = 25 embryos/biological replicate). Exact *p* values are indicated above (Welch's *t*-test) (Right). (**M**) Stain-free signal (Gürtler et al, 2013) of the gels employed as loading control for Western blot in panel (**L**). Source data are available online for this figure.

 © The Author(s)

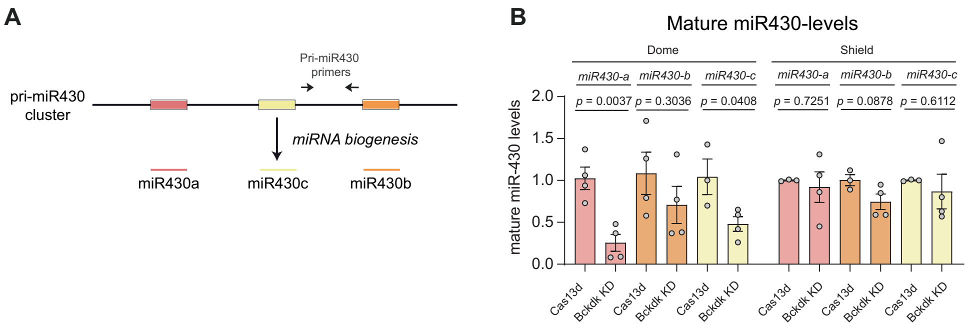

Figure EV7. *bckdk* mRNA depletion affects the processing of miR-430.

(**A**) Schematic representation of miR-430 triplet, RT-qPCR primer employed for measuring of primary miR-430 levels are indicated with black arrows (Adapted from Hadzhiev et al, 2023). (**B**) RT-qPCR analysis showing levels of mature miR-430 isoforms (miR430-a, red; miR430-b, orange; and miR430c, yellow) at 4.3 hpf (Dome) and 6 hpf (Shield). Results are shown as the averages ± standard error of the mean from three independent experiments with two biological replicates each ($n = 10$ embryos/biological replicate) for RfxCas13d protein alone and RfxCas13d plus 2 gRNAs targeting *bckdk* mRNA (see Methods for details). ncRNA *u4atac* was used as a normalization control. Exact p values are indicated above (unpaired t-test). Same data as represented in Fig. 4C.

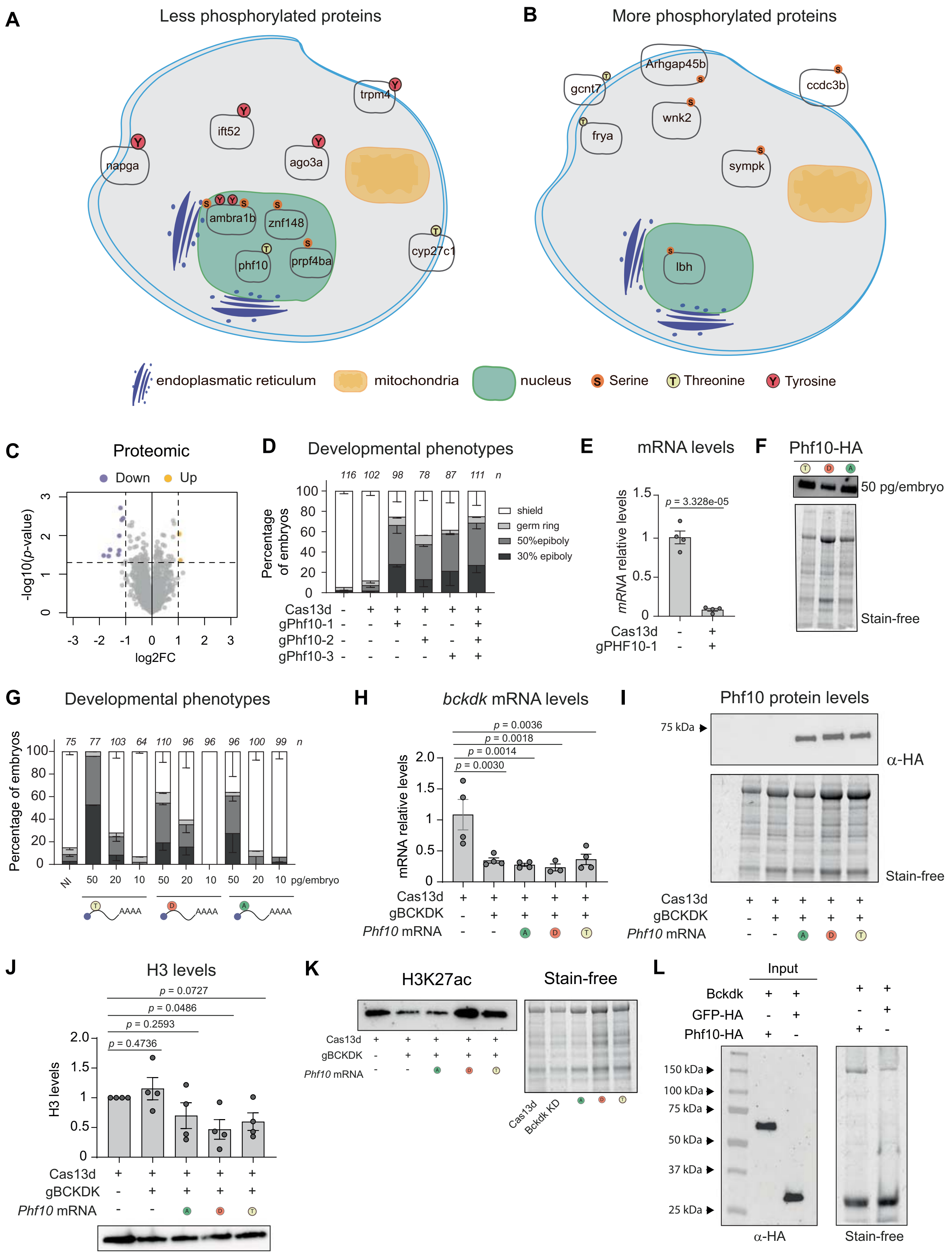
A
Less phosphorylated proteins
B
More phosphorylated proteins
endoplasmatic reticulum
mitochondria
nucleus
Serine
Threonine
Tyrosine
C
Proteomic
Down
Up
-log10(p-value)
log2FC
D
Developmental phenotypes
Percentage of embryos
shield
germ ring
50%epiboly
30% epiboly
Cas13d
gPhf10-1
gPhf10-2
gPhf10-3
E
mRNA levels
mRNA relative levels
p = 3.328e-05
Cas13d
gPHF10-1
F
Phf10-HA
50 pg/embryo
Stain-free
G
Developmental phenotypes
Percentage of embryos
pg/embryo
H
bckdk mRNA levels
p = 0.0036
p = 0.0018
p = 0.0014
p = 0.0030
mRNA relative levels
Cas13d
gBCKDK
Phf10 mRNA
I
Phf10 protein levels
75 kDa
α-HA
Stain-free
Cas13d
gBCKDK
Phf10 mRNA
J
H3 levels
p = 0.0727
p = 0.0486
p = 0.2593
p = 0.4736
H3 levels
Cas13d
gBCKDK
Phf10 mRNA
K
H3K27ac
Stain-free
Cas13d
gBCKDK
Phf10 mRNA
Cas13d
Bckdk KD
L
Input
Bckdk
GFP-HA
Phf10-HA
150 kDa
100 kDa
75 kDa
50 kDa
37 kDa
25 kDa
α-HA
Stain-free

 © The Author(s)

Figure EV8. Phospho-proteomic analysis upon *bckdk* mRNA depletion identifies Phf10 as a potential Bckdk target controlling MZT.

Schematic representation of the proteins found less (**A**) or more (**B**) phosphorylated upon *bckdk* mRNA knockdown condition. Its known cell localization (Bateman et al, 2022) and the target residues are represented. (**C**) Scatter plot representing the log2 fold change in protein level and the associated *p* value from four biological replicates ($n = 100$ embryos/biological replicate) at 4 hpf from embryos injected with RfxCas13d protein alone or with a mix of two gRNAs targeting *bckdk* mRNA, respectively. *p* values were calculated using a moderated *t*-test. Less (Down) or more (Up) abundant proteins are indicated in purple and yellow, respectively. Dashed lines indicated 2-fold in proteins levels and p value $= 0.05$. (**D**) Stacked barplots showing the percentage of phenotypes observed at 6 hpf from embryos injected with RfxCas13d (3 ng/embryo) alone or together with indicated gRNAs (1000 pg/embryo) targeting *phf10* mRNA. The results are shown as the averages ± standard error of the mean of each developmental stage from at least two independent experiments. The phenotype selection criteria were the same as in Fig. 1C. Number of embryos evaluated (n) is shown for each condition. (**E**) RT-qPCR analysis showing levels of *phf10* mRNA at 2 hpf in zebrafish embryos co-injected using a gRNA (gPHF10-1) targeting *phf10* mRNA and RfxCas13d protein. Results are shown as the averages ± standard error of the mean from two independent experiments with two biological replicates each ($n = 10$ embryos/biological replicate). *taf15* mRNA was used as normalization control. Exact *p* value is indicated above, unpaired *t*-test. (**F**) Western blot showing Phf10-HA expression at 6 hpf in zebrafish embryos injected with 50 pg/embryo of the *phf10-HA* mRNA versions used in Fig. 5. Stain-free signal (Gürtler et al, 2013) of the gel as loading control. (**G**) Stacked barplots showing the percentage of phenotypes observed at 6 hpf from embryos injected with 10, 20, or 50 pg/embryo of *phf10* mRNA WT (Phf10-16T) or modified in the residue phosphorylated by BCKDK. Phf10-16D (Aspartic acid, mimic the phosphorylation state mediated by Bckdk), Phf10-16A (Alanine, mimic a constitutively non-phosphorylatable version). The number of embryos evaluated (*n*) for each condition is shown. The results are shown as the averages ± standard error of the mean of each developmental stage from two independent experiments. The phenotype selection criteria were the same as in Fig. 1C. Number of embryos evaluated (*n*) is shown for each condition. (**H**) RT-qPCR analysis showing *bckdk* mRNA levels at 4 hpf in zebrafish embryos co-injected with RfxCas13d protein and two gRNAs targeting *bckdk* mRNA, either alone or together with different versions of *phf10* mRNA used in Fig. 5. T: threonine (wild-type version); D: aspartic acid (mimics the phosphorylated state mediated by Bckdk); A: alanine (mimics a constitutively non-phosphorylatable version). Results are presented as the mean ± standard error of the mean from two independent experiments, each with two biological replicates ($n = 10$ embryos per replicate). *taf15* mRNA was used as the normalization control. Exact *p* values are indicated above, one-way ANOVA). (**I**) Western blot showing Phf10-HA expression at 4 hpf in the rescue conditions indicated in panel (**H**) (Top). Stain-free signal (Gürtler et al, 2013) of the gel as loading control (Bottom). A representative western blot from two independent experiments of HA signal is shown under the indicated conditions. (**J**) Barplots representing H3 levels relative to total proteins as the averages ± standard error of the mean from four independent experiments under the rescue condition experiment similar to indicated in panel (**H**) (Top). Zebrafish embryos were collected at 4 hpf ($n = 15$–20 embryos/biological replicate) (ns non-significant. Exact *p* values are indicated above, Welch's *t*-test). Western blot showing H3 protein levels (Bottom). (**K**) Representative western blot image for H3K27ac of embryos co-injected with RfxCas13d protein and two gRNAs targeting *bckdk* mRNA, either alone or together with different versions of *phf10* mRNA described in panel (**H**). Zebrafish embryos were collected at 4 hpf ($n = 25$ embryos/biological replicate) (Left). Stain-free signal (Gürtler et al, 2013) of the gel as loading control (Right). (**L**) Western blot showing Phf10-HA and GFP-HA expression employed as input for the co-immunoprecipitation assay (Fig. 5H). Phf10-HA (50 pg/embryo) or GFP-HA (50 pg/embryo) were co-overexpressed with *bckdk* mRNA (200 pg/embryo). Stain-Free signal (Gürtler et al, 2013) of the gel as loading control (Right). Source data are available online for this figure.

© The Author(s)
